# Improving Oral Health in Older Adults and People With Disabilities: Protocol for a Community-Based Clinical Trial (Good Oral Health)

**DOI:** 10.2196/14555

**Published:** 2019-12-18

**Authors:** Jean Schensul, Susan Reisine, James Grady, Jianghong Li

**Affiliations:** 1 Institute for Community Research Hartford, CT United States; 2 University of Connecticut School of Dental Medicine Farmington, CT United States; 3 Department of Public Health Sciences University of Connecticut School of Medicine Farmington, CT United States

**Keywords:** oral health, elderly, oral hygiene, prevention, clinical trial, crossover design

## Abstract

**Background:**

Low-income older adults experience disparities in oral health problems, including caries and periodontal disease, that can exacerbate already high levels of chronic and acute health problems. Behavioral interventions have been shown to improve oral health status but are typically administered in institutional rather than community settings. Furthermore, multiple simultaneous interventions at different levels in the locations where people live and work are likely to have more impact and sustainability than single interventions in clinical settings.

**Objective:**

This paper outlines a protocol for conducting a bilingual 5-year community-based trial of a bilevel intervention that addresses community norms, beliefs, intentions, and practices to improve oral health hygiene of vulnerable older adults living in publicly subsidized housing. The intervention utilizes (1) a face-to-face counseling approach (adapted motivational interviewing [AMI]) and (2) resident-run oral health campaigns in study buildings.

**Methods:**

The study’s modified fractional factorial crossover design randomizes 6 matched buildings into 2 conditions: AMI followed by campaign (AB) and campaign followed by AMI (BA). The total intervention cycle is approximately 18 months in duration. The design compares the 2 interventions alone (T0-T1), and in different sequences (T1-T2), using a self-reported survey and clinical assessment to measure Plaque Score (PS) and Gingival Index (GI) as outcomes. A final timepoint (T3), 6 months post T2, assesses sustainability of each sequence. The intervention is based on the Fishbein integrated model that includes both individual and contextual modifiers, norms and social influence, beliefs, attitudes, efficacy, and intention as predictors of improvements in PS, GI, and oral health quality of life. The cognitive and behavioral domains in the intervention constitute the mechanisms through which the intervention should have a positive effect. They are tailored through the AMI and targeted to building populations through the peer-facilitated oral health campaigns. The sample size is 360, 180 in each condition, with an attrition rate of 25%. The study is funded by National Institute of Dental and Craniofacial Research (NIDCR) and has been reviewed by University of Connecticut and NIDCR institutional review boards and NIDCR’s clinical trials review procedures.

**Results:**

When compared against each other, the face-to-face intervention is expected to have greater positive effects on clinical outcomes and oral health quality of life through the mediators. When sequences are compared, the results may be similar but affected by different mediators. The arm consisting of the BA is expected to have better sustainability. The protocol’s unique features include the comparative effectiveness crossover design; the introduction of new emotion-based mediators; the balancing of fidelity, tailoring, and targeting; and resident engagement in the intervention.

**Conclusions:**

If successful, the evaluated interventions can be scaled up for implementation in other low-income congregate living and recreational settings with older adult collectives.

**Trial Registration:**

ClinicalTrials.gov NCT02419144; https://clinicaltrials.gov/ct2/show/NCT02419144

**International Registered Report Identifier (IRRID):**

DERR1-10.2196/14555

## Introduction

Older adults [1-7] and adults with disabilities [1] experience a high prevalence of tooth decay, periodontal disease, edentulism, unmet oral health treatment needs, and impaired oral health quality of life. Oral health is associated with systemic health problems [8] and chronic diseases of older adulthood. For example, declining cognitive function [9], dementia associated with serum *Porphyromonas gingivalis* (a causal pathogen for periodontitis), high immunoglobulin G [10], and xerostomia resulting from multiple medication use, cancer treatments, and diabetes are associated with poor oral hygiene, high levels of decay, tooth loss, and edentulism [11-13]. Periodontal disease is associated with heart attack and stroke [14-16], and periodontal treatment can improve control of diabetes mellitus [13]. In addition, poor oral health affects oral health quality of life [8].

There continue to be significant ethnic and racial, class, and medical disparities with respect to oral disease, oral health care, and oral health–related quality of life [7,17-19]. Oral health problems have a greater impact upon quality of life among older African Americans as compared with whites on every dimension and especially in psychological discomfort, pain, and functional limitations [7,20,21]. US and international bodies recognize the importance of addressing oral health through hygiene improvement [4,22] and Good Oral Health behavioral management [23-25]. There is general agreement that sustainable promotion of public health interventions requires a multilevel approach [26-29] and that multilevel approaches that include cognitive, social, behavioral, and norms change components are needed to reduce disparities in oral health and other systemic health conditions.

Improving oral health of older adults, especially low-income and racial and ethnic minority adults who also suffer from disproportionate rates of chronic diseases such as cardiovascular disease and diabetes, is a primary national and international priority [30]. The development of low-cost preventive interventions conducted in locations where older adults live can lead to potentially sustainable normative support for oral hygiene, locally tailored and targeted approaches, and ongoing positive changes in specific oral health practices (brushing, flossing, cleaning mouth and tongue, and cleaning dentures.). Such interventions can reduce short- and longer-term psychosocial and economic costs associated with debilitating oral health problems and may help to prevent exacerbation of chronic illness and disability.

The approach described in this protocol offers important innovations with respect to public health dentistry and preventive oral health interventions for older adults. Public health dentistry typically does not tackle multilevel approaches to community-based prevention, especially with older adults. The study protocol describes a bilevel approach in line with the recognition that multilevel interventions in public health have greater impact and sustainability than single interventions. Furthermore, it is based on a theoretical framework, the Fishbein integrated model (IM) supplemented by Bandura’s notions of self-efficacy and practice to mastery [31-33]. This approach includes constructs or mechanisms of change that are operationalized at both the individual and the group levels to improve knowledge, build pro oral health norms, reinforce self-efficacy and intentionality to engage in oral health self-management, and increase behavioral skills.

The study uses adapted motivational interviewing (AMI), a more structured approach to motivational interviewing (MI) with individuals. MI has been found useful in individual level oral health and hygiene interventions with adults [34,35]. AMI offers a more appropriate approach to public health interventions with older adults and adults with disabilities because it is partially scripted in advance, thus standardizing the operationalization of IM’s theoretical domains. The AMI intervention developed for this study tailors the intervention to individual needs [36].

**Figure 1:**
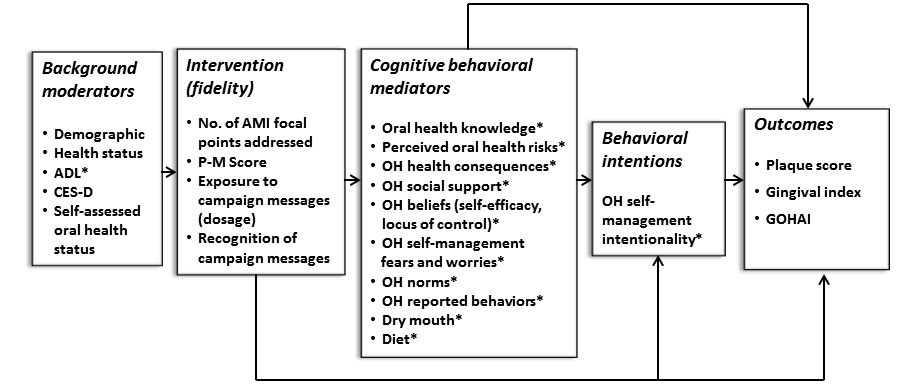
Good Oral Health study theoretical framework. ADL: Activity of Daily Living; AMI: adapted motivational interviewing; CES-D: Center for Epidemiologic Studies Depression; GOHAI: General Oral Health Assessment Index. OH: oral health. * indicates AMI focal points and campaign messaging.

This paper describes the study protocol for the community-based clinical trial entitled: Good Oral Health—*A Bilevel Intervention to Improve Oral Adult Oral Health.* The Good Oral Health study is a theoretically driven bilevel intervention to address oral hygiene self-management among older adults with limited resources, experiencing multiple health disparities (National Institute of Dental and Craniofacial Research [NIDCR] grant number DE24168). The study utilizes a crossover design to test a face-to-face counseling intervention against an interactive approach to change oral health norms and behaviors delivered through oral health fairs or campaigns. Both approaches target the same cognitive and behavioral domains as specified in the study’s theoretical model (see Figure 1) [37]. Other unique features of the study design are its attention to fidelity of implementation plus tailoring and targeting to individuals and groups and the engagement of peer educators as partners in the intervention. The primary outcomes are clinical assessment of Plaque Score (PS) and Gingival Index (GI), both standard measures for evaluating oral health interventions [38,39]. A secondary outcome is perceived oral health quality of life [8,40]. There is evidence that cognitive behavioral interventions can improve GI and PS in low-income populations [41-43]. This study is expected to show the positive effects of such an intervention with an understudied low-income population of vulnerable older adults including those with disabilities who are residents of subsidized senior housing and experience significant oral health disparities. Multimedia Appendix 1 provides a summary of the study protocol.

## Methods

### Study Design

The study is based on a pilot intervention that evaluated the results of an individualized face-to-face AMI intervention combined with a resident-managed oral health campaign in one rent-subsided older adult building in central Connecticut [44]. The intervention was successful in improving gingival health and reducing plaque, but the relative impact of each of the intervention components could not be evaluated because they were held simultaneously. The clinical trial protocol is designed to disaggregate and compare the individual and combined effects of the face-to-face intervention and the oral health campaign using a modified fractional factorial crossover design [45]. The design allows for disaggregating a pilot study that combined 2 interventions (AMI and oral health campaign) with positive results to enable an evaluation of which of the 2 components would have the best effect. Sequencing (systematic recombining), an element of the modified fractional factorial design (MFFD) is an efficient way of determining whether one combination or another has a better immediate and long-term effect. The MFFD design avoids the cost of multiple sites and control groups. The intervention activities are linked to the conceptual domains in the adapted Fishbein model (see Figure 1).

The primary study aims are to evaluate the 2 main components of the intervention, the AMI and the oral health campaign, against each other and in different sequences and to assess the mechanisms (norms, beliefs, attitudes, intentions, and practice) through which the intervention operates at each time point.

The study hypotheses include the following: (1) the face-to-face intervention component of the overall intervention (AMI) will produce better short-term clinical outcomes and changes in mediators than the oral health campaign component; (2) the oral health campaign component followed by the individual component will produce better midterm and long-term clinical outcomes than the individual component followed by the oral health campaign; (3) the 2 sequences will result in differences in changes in the mediating cognitive domains at midterm; (4) within the face-to-face component, exposure to more mediating domains will result in better clinical outcomes; and (5) both conditions will have an equivalent effect on the secondary outcome—oral health quality of life at end point.

**Figure 2:**
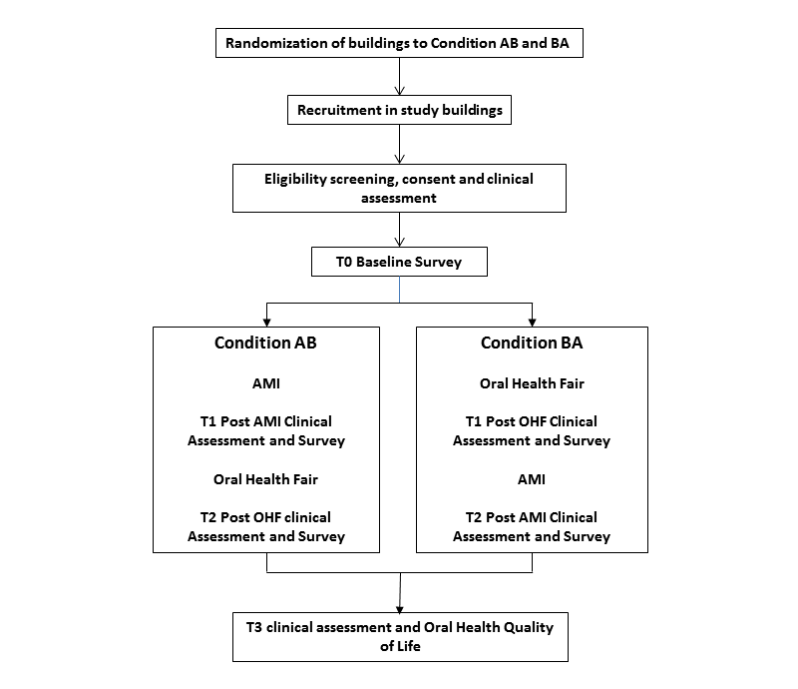
Good Oral Health study design. AB: adapted motivational interviewing followed by campaign; AMI: adapted motivational interviewing; BA: campaign followed by adapted motivational interviewing. OHF: oral health fair.

The study is a group randomized controlled trial (GRCT) in which 6 study buildings of between 125 and 375 apartment buildings housing independently living adults aged 62 years and older and people with disabilities are matched by size and randomized to one of 2 conditions (AMI followed by campaign [AB] and campaign followed by AMI [BA]), 3 buildings in each condition. In condition AB, the face-to-face intervention (A) precedes the oral health campaign (B) and in condition BA, the sequence is reversed. Buildings are selected based on size and geographic distance from one another; paired based on population age, ethnicity, and gender characteristics; and randomized by the study’s biostatistician. The procedure is described in the study protocol, which can be found on the study website [46]. Prior network studies in similar buildings [47,48] indicate that there is little or no communication among residents across buildings, and residents do not move from building to another. Thus, the risk of contamination is low. However, those who do move from one study building or condition to another will be eliminated from the study. The intervention is administered in 3 cycles of 18 months each, 2 buildings per cycle, 1 from each condition. Measures are taken at baseline (T0), after the first intervention (T1), after the second intervention (T2), and 6 months post T2 (T3); T0-T1 measures assess the impact of one intervention against the other, T1-T2 measures assess the impact of both interventions in different sequence, and T2-T3 evaluates outcome sustainability (see Figure 2).

### Study Site

The 6 study buildings are located in low-income areas of 3 Connecticut towns. The ethnic and racial composition of the population in these buildings consists of approximately 40% African American/Caribbean; 45% Latinos (mainly Puerto Ricans); and 10% to 15% other residents, mainly of European American or South/South East Asian origin. The rate of people with disabilities ranges from 15% to over 40%. To participate, buildings must have a common space and private locations for surveys and clinical assessments. Memoranda of agreements are signed with the managements of all buildings to guarantee participation and exempt building management from responsibility for any adverse intervention consequences. The study population speaks English, Spanish, or both. All materials used for recruiting, consenting, survey administration, and implementation of both intervention approaches are translated from English to Spanish and back translated to ensure that meaning in the 2 languages is preserved. Recruitment and all interventions are conducted in both languages or either language based on the participant’s choice.

### Study Participants and Sample Size

The study population includes 360 residents, approximately 60 from each building, totaling 180 in each condition. The expected attrition rate over 4 time points is 25%. The inclusion criteria include (1) being aged 18 years and older, (2) having permanent residence (6 months or more) in study buildings, (3) living without conservator, (4) judged competent to give informed consent by responding correctly to 5 simple questions about the study during the consent process, and (5) 2 or more natural teeth. The exclusion criteria include (1) being temporary or short-term building resident; (2) being under conservatorship, (3) cognitively unable to give informed consent or respond to 3 to 5 questions about the study, (4) being edentulous; (5) having a history of infective endocarditis, prosthetic cardiac valve replacement in past 6 months, or insertion of an arterial stent or myocardial infarction in past 6 weeks or being on dialysis. The study is approved annually by the University of Connecticut Health institutional review board (IRB) and by NIDCR and Rho consultants.

### Study Recruitment

Recruitment takes place during the first 5 to 6 months of each 18-month intervention period in each cycle. Recruiters are bilingual and of diverse ethnic and racial backgrounds, matching the backgrounds and languages of most participants. Steps in recruitment include initial discussions with building management, tenant associations, or other internal administrative bodies; 2 formal presentations to building residents, followed by presence on site several times a week; and informal engagement with residents and hosting of informal events, gatherings, and refreshments in community rooms and on building floors throughout the recruitment period. Public events sponsored by the study are conducted in communal spaces that can accommodate individuals with disabilities or in wheelchairs.

Participants found to be eligible are contacted within 2 to 3 days for an appointment to obtain informed consent and to conduct the clinical assessment. At the appointment, they are rescreened, informed about the study, and written consent is obtained. Consent forms are read to participants in English or Spanish, regardless of reading ability. Participants who are unable to read give verbal consent, which is recorded by the consenter and witnessed by a second member of the study staff. The signatures of both are recorded on the form with the participant’s name. Ineligible participants are invited to join an *oral health campaign* committee in their building.

Once consented, participants in the study are clinically assessed after which they are scheduled for a survey within the next 2 weeks. Retention is encouraged through continuous staff presence in the intervention buildings, phone calls and face-to-face reminders, home visits, casual encounters, flyers, face-to-face communication, and study-hosted social events such as bingo and ice cream socials throughout the study. These methods have been found to be useful for retention in prior studies on other health topics with similar populations in subsidized senior housing [29,44,49]. After completion of these steps, participants are engaged in scheduled one-on-one sessions (AMI) or for participation in oral health fairs, depending on the building. One month after having completed each of these interventions, participants complete a survey and clinical assessment (T1) and enter into the second phase of intervention. This is followed by a third survey and clinical assessment (T2) 1 month after completion of the second intervention. Furthermore, 5 to 6 months after the third evaluation point, participants receive a final clinical assessment and repeat the oral quality of life scale (T3).

### Interventions

The 2 interventions are conducted orally and through demonstrations to accommodate those with limited literacy. Participation does not require the ability to read.

#### Condition 1 (Adapted Motivational Interviewing Followed by Campaign): Adapted Motivational Interviewing With Individual Participants

The individual-level intervention (AMI) is based on a successfully piloted 45-min adapted motivational interview protocol [44] administered in English or Spanish by trained African American and Latino interventionists. It takes place within 2 to 4 weeks after completing the most recent survey (baseline or T1 depending on the condition). AMI intervention tailoring is based on 5 key elements: (1) an initial focused conversation with the participant about issues of concern, (2) responses that are below the cutoff point on the 12 mediator domains in the study model (see Table 1), (3) a review of the plaque scoring sheet that illustrates each person’s deposition of plaque to help target brushing, (4) a brushing flossing skills assessment using a typodont and videos on brushing and flossing, and (5) a written and signed plan of action. A formula determines whether a participant falls above or below a designated cutoff point for each domain. Any participant who falls below the cutoff point on any domain receives an intervention for that domain. The domain cutoffs for each participant are calculated based on the most current survey: in condition AB, they are calculated on the study baseline survey, and in condition BA, they are calculated on the T1 survey.

Cutoffs were developed during the pilot study by deciding whether domain mean scale scores or individual scale or index items were the best indicators of intervention need. These decisions are summarized in Table 1. A software formula calculates individual cutoffs and transfers the results to an Access data form that documents the AMI intervention. Facilitators use this form to prepare their intervention material by first identifying domains below the cutoff that require intervention followed by checking the survey results for those domains to identify specific items with incorrect responses. Domains and items that need attention are recorded on a *focal point checklist* for use in the intervention.

**Table 1:** Domain scales and cutoff scoring.

Domain		Scale description	Cutoff points
1. Activity of daily living		8 items with response categories 0 (no help needed); 1 and 2 help needed.	Need help on any of these
**2. Oral health knowledge**		7 items, true/false (Items in both scales are 4-point Likert scales from 1=strongly disagree to 4=strongly degree)	<5 correct
	3b. Oral health self-efficacy	5 items	>Mean of items <3 (disagree and strongly disagree)
	4b. Locus of control	7 items (only 1 considered for cutoff)	If response to single item was agree or strongly agree
3. Oral health norms: beliefs about importance of oral hygiene		9 items, Likert scale with 4=very important and 1=not at all important.	1 or 2 on any item (not at all important, not very important)
4. Oral health social support		4 options (Likert scale with 0=no and 1=yes)	If all sources are *0* (none)
5. Oral hygiene behaviors		6 options (Likert scale with 1=never and 6=more than twice a day)	Brushing: <2 times per day; flossing: <1 time per day
6. Perceived oral health risks		5 questions, 4-point Likert scale	Mean <3 (4=very unlikely, 3=unlikely, 2=likely, 1=very likely)
7. Self-management worries		23 questions, 4-point scale, 1=very and 4=to not at all	Mean <3 for scale (4=not at all, 3=not much)
8. Self-management fears		5 questions, 4-point Likert scale, 1=very to 4=not at all	Mean <3 (4=not at all, 3=not much)
9. Oral health self-management intentionality		10 items, 3-point Likert scale (0=no, 1=some, 2=good possibility)	Mean <1 (0=no possibility, 1=slight possibility)
10. Dry mouth		8 yes/no questions	Yes to at least one question
11. Diet		5 items, 5-point Likert scale from never to >5 times daily.	>2-3 times a day on any item
12. Plaque Score/Gingival Index		Range from 0 to maximum of approximately 192 for each.	Mandatory for All participants regardless of score.

To prepare for each AMI administration, the intervention facilitators create a file in advance, consisting of the completed intervention focal point check list, duplicates of a plan of action form, and the most recent clinical assessment showing the distribution of plaque.

The AMI is conducted in a private location in each building. Interventionists are required to address brushing and flossing and a minimum of 2 to 3 other cognitive or behavioral mediators. They record elements of the patient narrative in the Access data form and discuss each focal point with the participant using an interactive dialogue approach and referring to a standard script to correct misunderstandings, expand knowledge, and explore barriers to intention (knowledge, beliefs, and attitudes). To target brushing and flossing, interventionists show participants the results of their most recent plaque assessment and brief videos demonstrating techniques for brushing and flossing teeth. Participants then practice on a typodont and are scored for brushing, flossing, and denture cleaning techniques using a standardized skills assessment checklist, and scores are calculated from 1 to 4 for each (1=lowest, and 4=highest). The scoring is repeated until the participant achieves maximum improvement (practice to mastery), and the final score is recorded as a process evaluation data point. In the final step, interventionists review the discussion with the participant and help the participant to build a plan for addressing the main cognitive domains that impede their own oral health self-management and improving oral health hygiene behavior. Participants receive a copy of their plan, and a duplicate is kept in the file along with the focal point checklist, the Access focal points and PS data sheets and the skills assessment scored sheet. AMI administrations are audio recorded as a quality check with consent. Tailoring is accomplished through directing interventionist comments to the concerns raised in the participant’s opening narrative and the specific domains that require attention because of their low scores.

Fidelity to the AMI protocol is achieved through annual trainings, observation, and feedback on AMI delivery in each cycle. All AMI sessions are audio recorded with permission of the participant, and 10% of the recorded sessions are reviewed in every cycle in English and Spanish. Research charts are reviewed before filing to ensure case documentation such as recording cutoff domains, referral to a general study-approved script to address the domains, maintaining a hard copy and digital records of participant responses to domain-related discussions, participant concerns, and the participant plan. Furthermore, 10% of the research charts are reviewed every 6 months to ensure that intervention forms are complete.

#### Condition 2: Resident-Assisted Oral Health Campaign

The oral health campaign consists of 3 oral health fairs co-organized by a trained volunteer team of building residents (the campaign committee) that collaborates with bilingual study interventionists. The campaign protocol is based on the IM cognitive behavioral theory that guides the study, and processes derived from communications theory [50,51]. It is modeled after other tailored/targeted theoretically based large-scale communication interventions, scaled to fit the constraints of public housing settings. The local campaign model utilizes a group norms approach that relies on the social influence of a collective of motivators (in this case, residents and members of the intervention staff) [40] rather than a peer led diffusion-of-information model [52]. With this approach, recruits may be, but do not have to be identified as peer or opinion leaders to be included. Each committee consists of approximately 6 to 8 members, all screened ineligible for the study.

Efforts are made to attract both men and women representing the general pattern of diversity in each building for the campaign committee. As contributing participants in the study, they are asked to sign the IRB-approved consent forms agreeing to their participation as committee members. A small gift certificate and certificate of accomplishment are given to each committee member at the end of the campaign sequence in a public setting.

Committee members undergo a 12-session training program (see Table 2) of approximately 1.5 hours each, and makeups are possible for members who have missed a session. The curriculum is available on the study website [46]. The study staff provides refreshments at each session. Subsequently, staff and campaign committee members work together to organize and staff tables at each campaign event, representing each of the 12 domain messages.

**Table 2:** Oral health campaign curriculum.

Sessions	Description
Session 1	Orientation and group identity
Sessions 2-3	Introduction to Good Oral Health campaign; protecting and respecting other residents
Sessions 4-5	Learning about oral health and hygiene self-management; creating a campaign event schedule
Sessions 6-7	Creating an oral health campaign plan
Sessions 8-9	Developing theoretically based materials and messages
Session 10	Preparing and practicing for campaign event
Session 11	Finalizing campaign posters
Session 12	Finalizing and practicing campaign roles and activities (including scripts for facilitating discussion at session tables, welcome station, passport administration, and signup sheets

All residents in each building are invited to each of the campaigns, and they sign in at the door. Each attendee receives a passport that includes a space to check their presence at each message table. The passport records their name and exposure to and evaluation of each table visited. These data are entered into a computer database and provide (1) overall attendance of enrolled participants at each of 3 sessions, (2) unenrolled participants who attend each session (reach), (3) dosage (number of tables each participant visits at each campaign), and (4) whether they liked or did not like their experience at the table (acceptability). Passports of participants are placed in their research charts.

All oral health fairs are required to include the following:

1. Bilingual message tables, 1 per theoretical domain, with messages, games, activities, and/or handouts staffed with bilingual study staff and at least one campaign member.
2. A 15- to 30-min bilingual presentation by the dental hygienists on general issues of importance in oral health and hygiene maintenance.
3. A question-and-answer period in which members of the audience can raise questions with the speakers on any topic related to oral health in the language of their choice.

Resident members of the Campaign Committee and research staff are stationed at each domain table designed to foster discussions with each participant based on their questions, issues, and concerns related to the domain assigned to the table (eg, diet, worries about self-management, perceived risks associated with poor oral health, brushing, and flossing). These activities are followed by snacks, music, and prizes.

### Sample Size and Power

Sample size calculations were based on the primary outcomes of continuous measures of GI and PS using estimates of effect and variation from a pilot grant. The MFFD design has 2 sequences (AB and BA) and 2 periods (1 and 2). Power was based on the first period, which conceptually can be considered a 2-arm parallel design with cluster randomization at the building level. Assuming a typical intraclass correlation in the range of 0.01 or 0.02, a design with 6 clusters of n of 56 for each condition would have effective sample sizes of approximately 160 to 220, respectively [53]. With n of 153 per group, our study has more than 95% power to detect a priori identified clinically meaningful mean differences.

### Measures

The study evaluation design includes intervention moderators, mediators, process variables, and outcome measures. All alpha coefficients are from the study’s baseline data.

#### Primary Outcomes (Clinical Assessments)

The primary outcomes are obtained through clinical assessments of oral hygiene status. The PS is a plaque scoring scheme developed by O’Leary et al [38] consisting of dichotomous presence or absence scores for bacterial plaque on each of 6 tooth surfaces using erythrosine disclosing solution. The nontoxic vegetable-based solution is applied to the teeth by the examining hygienist. The number of surfaces stained red is calculated over the total number of surfaces, and PS is expressed as a percentage of surfaces with plaque or a ratio. The GI [39] assesses the gingival status related to 6 surfaces of each tooth. Each surface is scored for gingival inflammation: 0=no visual signs of inflammation, 1=slight change in color and texture of the gingiva but no bleeding, 2=visual sign of inflammation and bleeding upon swiping, and 3=overt inflammation and spontaneous bleeding. The index is calculated by summing each surface GI and dividing by the total number of surfaces (mean value). Individual scores are summed to obtain a mean.

#### Secondary Outcome Measure

The *General Oral Health Assessment Index* (GOHAI) measures oral health quality of life. A commonly used 12-item measure, it was initially developed for older adults and has been used with low-income populations [54]. Responses for each statement are Likert scales ranging from 0 (always) to 4 (never); 3 items are reversed coded. Response codes are summed across the 12 statements to give a 0 to 48 overall score (Cronbach alpha coefficient=.801).

#### Intervention Mediators

##### Oral Health Knowledge

Oral health knowledge is a 7-item true/false test based on a previously developed knowledge test used with low-income older African Americans [55] (Cronbach alpha coefficient=.66).

##### Perceived Oral Health Risks

Perceived oral health risks consists of 5 questions asking about the chances of getting cavities, cancer, toothache, gum problems, and hospitalization because of an oral health problem, on a 4-point scale from very unlikely (4) to very likely (1; Cronbach alpha coefficient=.761).

##### Oral Health Social Support

Oral health social support is assessed with 2 questions developed in the pilot study [44]:

1. Who do you go to for health information in this building (check all that apply)? The responses are no one, other residents, building management, and people who come to provide services in the building. The scores range from 0 to 4.
2. How many residents do you talk to if you need information about health problems and how to handle them, not counting the building managers or others who work there? The responses are no one, 1 to 2, 3 to 4, and 5 or more. The scores range from 0 to 3.

##### Oral Health Beliefs

Oral health beliefs include 2 subscales of the Dental Coping Beliefs Scale [56-58]; *Self-efficacy* and *Locus of Control*. The *Self-efficacy* scale consists of 5 items, and *Locus of Control* is measured with 7 items Responses to items for both subscales are 4-point Likder scales ranging from strongly agree (4) to strongly disagree (1) adapted from the study by Sherer et al [59]. Self-efficacy scores range from 5 to 20, with higher scores indicating higher self-efficacy (Cronbach alpha coefficient=.603). Locus of control scores range from 7 to 28, with higher scores indicating lower external locus of control (Cronbach alpha coefficient=.72).

##### Oral Health Self-Management Fears and Worries

These are 2 new scales that include items identified by residents in focus groups, related to the topic. Both scales were piloted with good results during the pilot study phase (Oral Health Self-Management worries: Cronbach alpha coefficient=.90; Oral Health Self-Management fears: Cronbach alpha coefficient=.75). The *Oral Health Self-Management Worries Scale* consists of 23 items focused on worry or embarrassment related to taking care of teeth, mouth, and dentures. Approximately one-third of the study population overall has dentures. Responses are on a 4-point scale ranging from (1) very worried to (4) not at all worried. Scores range from 23 to 92, with higher scores indicating less concern (Cronbach alpha coefficient=.91). The *Oral Health Self-Management Fears scale* consists of 4 items about fears of the health consequences of not caring for teeth and gums. Items are assessed on a 4-point scale from (1) very to (4) not at all. Scores range from 4 to 16, with higher scores indicating less fear (Cronbach alpha coefficient=.82).

##### Oral Health Norms

Oral health norms (perceived importance of actions) is measured with a 9-item scale that assesses the perceived importance of oral hygiene behavior from (very important 4) to not important at all (1; Cronbach alpha coefficient=.688).

##### Oral Health-Reported Behaviors

Questions on frequency of brushing teeth, flossing teeth, and cleaning dentures. Responses to each are never (0), once a week (1), a few times a week (2), once a day (3), twice a day (4), and more than twice a day (5).

##### Oral Health Self-Management Intentionality

It uses the protocol described by Ajzen and Fishbein [60] and Tedesco et al [61,62], adapted for the clinical trial based on pilot data. Participants rate the possibility (likelihood) of performing 10 oral health behaviors on a 3-point Likert scale (0=no possibility to 2=good possibility).

##### Dry Mouth

It is an index consisting of 8 questions with yes/no responses related to indicators of dry mouth adapted from a study by Gerdin et al [63]. Responses are yes (1) and no (0).

##### Sugar Intake

It is an index that consists of 5 questions related to the consumption of sweet or starchy foods with responses including never (0), once in a day (1), 2 to 3 times a day (2), 4 to 5 times a day (3), and more than 5 times a day (4; Cronbach’s alpha coefficient=.619).

#### Intervention Process Variables

Intervention process variables include the number of focal points addressed in the intervention based on the total number of scores below the cutoff points for main cognitive and behavioral domains measured and addressed in the intervention; exposure to norms-based campaign measured with dosage (record of presence at event); and survey questions that record post exposure recognition of campaign messages including recalled participation in fairs, recognition of logos and messages, and perceived impact of fairs on self-management.

#### Additional Measures

##### Demographic Background Information

Items include age, gender, building, length of time in building, length of time in United States, marital status, race and ethnicity, current living arrangement, times moved in the past year, work status, religious engagement, income and income satisfaction, language use, telephone, transportation availability, home care, and health/dental health insurance.

##### Activity of Daily Living

Activities of daily living (ADLs) is a widely used measure of the physical functioning status of an individual. It consists of 8 behaviors that indicate ability to take care of personal basic needs [64,65]. The responses are 0 (no help), 1 (need some help), and 2 (unable to do activity even with help).

##### Center for Epidemiologic Studies Depression Scale Short-Form

Center for Epidemiologic Studies Depression (CES-D) Scale Short-Form is a 10-item short version of the CES-D screening instrument that measures depressive symptoms in community populations. The Spanish version of the 10-item CES-D has been validated for use with Puerto Rican older adults and used in several studies of older adults, including senior residents of public housing in the study area [66-68]. Responses are no=0, yes=1 and scores range from 0 to 10, with a higher score indicating more symptoms of depression (Cronbach alpha coefficient=.631).

##### Health Status

It consists of two indices: (1) an index assessing current health status based [66] on self-reported diagnosis of 13 physical health problems common in older adults and (2) an index of physical health distress based on whether each physical problem is perceived as preventing normal participation in daily activities. Responses are yes/no to each item. Responses are summed for each variable.

##### Self-Assessed Oral Health Status

It is a single 4-point Likert scale of subjective oral health status [40], ranging from poor (1) to excellent (4).

### Data Management and Quality Checks

All survey data are collected using the Questionnaire Development System (QDS) [69] software and face-to-face administration. Once the interviewer or other field team member realizes a potential data entry error, immediately after the interview, he or she will notify the data manager to double check the entered responses and will correct any error in QDS Data Warehouse. The data manager may also identify key variable entry errors during the data management process. All changes are automatically documented and tracked for quality control. The data are uploaded into QDS Warehouse in batches and cleaned and amalgamated into a data analysis database for each cycle. In addition, 3 separate databases collect all participant tracking and intervention and clinical assessment data. These data are converted to a spreadsheet and merged into the master data analysis survey database by cycle. Data files for the 4 time points are integrated first for T0-T1, then for T0-T1-T2, and finally for T0-T3 to allow for longitudinal short, intermediate, and long-term analyses. AMI intervention data quality is checked by reviewing the first 5 files and an additional 5 files chosen randomly per cycle in hard copy and audio files in English and Spanish. Survey quality checks are conducted every 6 months to review missing data or errors in entry. Records are kept of any changes in data analysis files related to routine cleaning, and additional records are kept for variable recodes, new variable construction, and outliers during the analysis of the baseline data and subsequent time points.

### Data Analysis

A comprehensive statistical plan addresses the modified fractional factorial crossover design. The usual inspection for outliers and influential data points is conducted along with summary statistics and evaluations of distributions of the data. In the case of nonnormal data, we use standard transformations (eg, log transformations) or explore alternatives (eg, nonparametric approaches or other distributions such as Poisson) or create categorical or nominal variables.

For period 1, the parallel-arm phase, standard approaches for a parallel 2-arm randomized study are conducted for the first period of the sequence, across all buildings. This analysis allows direct comparison of the AMI versus the campaign approach for clinical outcomes (study hypothesis 1). For periods 1 and 2, assessing sequence of interventions, study hypothesis 2, which sequence gives better clinical outcomes, are addressed using repeated-measures models. Depending on whether the outcome is continuous or dichotomous, we will use general linear mixed models or general estimating equations to fit a model with intervention and period effects using the MIXED procedure in SAS. Each set of measures from the same person (eg, gingival outcomes) is treated as a correlated cluster of data. An auto-correlation structure of 1 is likely to be appropriate. Customized contrasts can be constructed to make comparisons between time points. Time-varying covariates can be used in these models, and some missing data (under missing at random or missing completely at random assumptions where applicable) are allowable.

For baseline and health status variables, as this study randomizes at the level of site and individuals are not randomized, we will stratify some results (or adjust in models) for demographics such as age, gender, and marital status and individual characteristics such as health status, ADLs, depressive symptoms (CES-D), and oral health status (self-assessment). Any variable that shows potential for confounding or effect modification will be accounted for appropriately in all stages of the analysis.

For intervention variables, as all subjects are exposed to the same interventions, we will unpack the intervention into (1) dosage (percentage of talking points covered for all focal points —domains—addressed in the intervention and (2) exposure to focal point messages during campaigns. Study hypothesis 5 regarding dosage of interventions will be assessed by creating an ordinal scale that measures an individual’s exposure to talking points through participation in the AMI and exposure to messages via campaigns, thus creating a measure of dosage. We will then assess if dosage predicts better outcomes. This can be done directly (eg, compare mean gingival scores across dosage levels with analysis of variance) or dosage can be used as a predictor variable in the statistical models and causal pathway analyses. Practice to mastery (a 4-point Likert scale) and focal points (count variable) can be analyzed using similar techniques and approaches.

To address the secondary outcome of GOHAI, we will conduct a set of secondary analyses with GOHAI as a continuous outcome. It will also be incorporated into our statistical/mediator models as a predictor to see if it acts as an effect modifier.

To address cognitive mediators, the following variables will be incorporated into the causal pathway analysis to address example hypotheses 3 and 4: knowledge, oral health beliefs/norms, social support, oral hygiene behaviors, fears/worries, practice to mastery, and behavioral intentions. We will use longitudinal mediation to compare the two conditions (AMI followed by oral health campaign against oral health BA) in terms of whether they differ significantly in the amount of change that they engender in the GI and PS, indirectly through the mediators measured in our model.

We are interested in examining whether there is a change in our mediators over time, and if so, whether this change significantly leads to a change in behaviors, as assessed by the GI and PS. Recent methodological studies actually show that the analysis of change is more appropriately captured by specifying change (differences) in self-efficacy and our other mediators and behavioral outcomes between adjacent time points (ie, T2-T1 and T3-T2) as latent difference scores [70-73], and explicitly modeling these change scores to represent dynamic change, that is, the impact of change in the mediators on change in the behavioral outcomes.

Hypothesis #3 will be tested using latent change score analysis specifying AMI and campaign as predictors of change in behavioral beliefs (ΔBB), OH social support (ΔSS), locus of control beliefs (ΔCB), self-efficacy (ΔSE), fears/worries (ΔF/W), social norms (ΔSN),practice to mastery (ΔP2M), and behavioral intentions (ΔBI) from baseline to first and second follow-up.

To test hypothesis 4, we will conduct dynamic mediation using latent change (difference) score mediation analysis. The analyses will be conducted in Mplus 7, where change (difference) scores representing differences in each mediator and outcome variable from baseline to final follow-up that is, T2-T1 (ΔT_21_) and T3-T2 (ΔT_32_) will be generated. It is the difference scores of the mediators and outcome variables that are modeled. Generally speaking, causal paths specifying latent (true) change, represented by change in knowledge, oral health beliefs/norms, oral health social support, oral hygiene behaviors, fears/worries, practice to mastery skills, and behavioral intentions in going from T1 to T2 and T2 to T3 will be specified, to predict change in GI and PS at T3 (ie, ΔGingival index_32_, ΔPlaque score_32_). To assess the impact of the change in self-efficacy (ΔSE), on changes in GI scores (ΔGingival index), direct and indirect paths from change in self-efficacy from baseline to first follow-up (ΔSE_21_) that predict the change in GI at the final follow-up (ΔGingival index_32_) will be specified and tested. Select direct and indirect paths from change scores of the other mediator variables to change scores of behavioral outcomes will also be specified in a similar fashion, and all these relationships will be tested simultaneously [74].

## Results

This study was funded in 2014, recruitment was initiated in mid 2015, after extensive reviews of the study, protocol and human subjects materials, as well as a full NIDCR 2 day site visit, as is normal for a U grant or cooperative agreement. All data collection was completed in July 2019, and analysis of the baseline data began in April 2019. Since the study is a clinical trial, the funders did not approve consideration of study data till all intervention data were collected by January 2019, to avoid any potential biasing of results. One paper summarizing the baseline association of moderators with study outcomes is in press, and another under review. Other papers are in process on the association of mediators with outcomes at baseline, changes from baseline to the first post assessment, evaluating one intervention approach against the other and diffusion effects of the oral health campaign to nonattenders.

## Discussion

Approximately 330,000 seniors or 16% of all public housing residents in the United States live in subsidized public housing and another 500,000 live in subsidized Section 8 housing [75]. As a place-based intervention, the study offers a model that could conceivably reach over 20% of all low-income older adults in the United States in some form of publicly supported congregate housing. Adults with disabilities in publicly subsidized senior housing constitute an additional reachable population. Thus, the proposed intervention has the potential to improve oral health and hygiene in a population of older adults and adults with disabilities who have limited access to dental care and who are often left out of public health and prevention programs.

The study’s modified fractional factorial approach disaggregates 2 components of a successful pilot intervention without the added expense of a control. Its comparative-effectiveness, crossover design allows for a comparison of the 2 primary components separately and together in different sequences. Finally, unlike many interventions, it adds a long-term sustainability component to evaluate which sequence has better sustaining power with respect to clinical and secondary outcomes. The GRCT design is implemented under maximally controlled conditions in a community setting, making it a model for community-based clinical trials.

Residents of low-income senior housing across the nation are very diverse. This protocol offers a bilingual approach that is culturally and individually tailored. All materials and research tools are developed, translated, and back translated for use in both English and Spanish, consistent with recommendations in the National Institutes of Health 2009 conference on interventions to improve oral health [76], and the interventions are tailored or targeted. The individual intervention is tailored to the social, clinical, and psychological needs of the individual based on scores on scales measuring mediating mechanisms. Targeting in the oral health campaign component is made possible through peer involvement in designing theoretically driven oral health messages and participants’ engagement with staff intervention experts and peer educators around mediators and oral hygiene skills. The theoretical model also encourages intervention fidelity by tailoring campaign information to each of the theoretical mediators. The engagement of building resident volunteer peers in development and implementation of the oral health campaign introduces practices known to be effective in communications interventions. These include delivery of messages through respected peer educators as well as expert role models, the active engagement of participants in games and other interactive learning experiences to enhance adult learning, the development and delivery of theoretically driven messages based on peer educator understanding of the language and concerns of their peers and other residents, and the development of resident capability to dispense information and instructions on appropriate oral health self-management.

The study is also unique in its use of clinical assessments and biomarkers in a community residential setting. These assessments are cost-effective and have implications for clinical and research programs in other community settings. Finally, emotions are now understood to play a role in prevention and treatment adherence [77]. Pilot work demonstrated the centrality of worries in oral health and hygiene self-care; however, there is no measure of oral health self-management worries. Pilot study data were used to create a new measure of oral health and worries that has promise as a predictor in that study and will be further tested in this clinical trial as a potentially valuable emotions-based predictor of poor oral health hygiene.

The intervention protocol should show potential for making much needed significant improvements in oral health behaviors of older adults (aged 62 years and older) and younger adults with disabilities (primarily aged 50-62 years) in publicly supported senior residences, especially as these rent-supported buildings now include up to 40% people with disabilities, an often unreached low-income population. Both interventions also can be implemented in senior centers or other places where older adults and people with disabilities gather, such as federally or state-funded lunch programs, and the AMI can be implemented easily and inexpensively at home as well. All of these opportunities can help to reduce the long-term need for acute dental care and treatment and can positively impact on diabetes and cardiovascular disease, which are problems associated with poor oral health in both of these vulnerable low-income populations.

## Appendix

Multimedia Appendix 1 Slide presentation detailing protocol components.

Multimedia Appendix 2 Peer-reviewer report from the National Institute of Dental and Craniofacial Research.

## Abbreviations

AB: adapted motivational interviewing followed by campaign
ADL: activity of daily living
AMI: adapted motivational interviewing
BA: campaign followed by adapted motivational interviewing
CES-D: Center for Epidemiologic Studies Depression
GI: Gingival Index
GOHAI: General Oral Health Assessment Index
GRCT: group randomized controlled trial
IM: integrated model
IRB: institutional review board
MFFD: modified fractional factorial design
NIDCR: National Institute of Dental and Craniofacial Research
PS: Plaque Score
QDS: Questionnaire Development System
