# Supplementary material for: Improving Oral Health in Older Adults and People With Disabilities: Protocol for a Community-Based Clinical Trial (Good Oral Health)
Source: JMIR Res Protoc. 2019 Dec 18;8(12):e14555. doi: 10.2196/14555 (PMC6939248; doi:10.2196/14555)
Supplement: Multimedia Appendix 1 [file resprot_v8i12e14555_app1.pdf]

Site Initiation Visit Agenda

GOOD ORAL HEALTH:
A BI-LEVEL INTERVENTION TO IMPROVE OLDER ADULT ORAL HEALTH
PROTOCOL NUMBER: 14-046-

Schedule - Day 1

- Welcome
- Introductions/Roles & Responsibilities
- Investigator Responsibilities
- Safety: Definitions, Collection, Reporting
- Protocol Overview
- Investigator Site File Review

II. Roles and Responsibilities: SDM and ICR

- **SDM**: Official contact with NIDCR; maintain regulatory binder; manage and submit budgets; all reporting; supervision of dental hygienists; provision of expert dental education; overall project oversight with ICR PI; IRB review and updates; conceptualization, data analysis, papers and presentations.

II. Roles and Responsibilities: SDM and ICR

- **ICR**: regular oversight of field operations; oversight of data collection, storage and synthesis, coordination with SDM and dental hygienists, collection and management of all study evaluation data; implementation of interventions; dissemination of data to community; presentation at conferences, data analysis, papers, presentations

II. Responsibilities of Study Staff

Interventionists:

The interventionists will be trained in the creation and implementation of the tailored AMI intervention and in working with residents to implement their oral health plan. They also will be trained to administer and score the oral health skills assessment (practice to mastery). Interventionists will also implement the campaigns with building residents.

The intervention coordinator will be responsible for coordinating and scheduling and ensuring quality administration of the tailored AMI intervention and the campaigns and storing and integrating fidelity data for the intervention (focal points, implementation plan and practice to mastery pre and post data). (See Campaign Trainers)

Survey Administrator: The survey administrators will be trained in administering consents, and the survey, saving the QDS files to a backup file in the field and uploading the files to a centralized server at ICR. The survey coordinator (evaluation field coordinator) will be responsible for coordinating the collection and integration of clinical assessment, and skills assessment data,

Responsibilities of Study Staff

Campaign Trainers: Campaign trainers will deliver the campaign curriculum and work with building residents to develop campaign messages and materials for the campaigns (see above for interventionists).

Expert Dental Educators/Supervisor Trainers. Ensure calibration of clinical assessment protocols and P to M; oversight of clinicians, provision of expert education at Campaign events

Dental Examiners: Hygienists will serve as dental examiners and conduct the clinical assessments. They also will be responsible for assuring that clinical data are properly recorded.

Research Assistant/Recruiters: The research assistant/recruiter will assist with recruitment, survey administration, note taking during project and Campaign Committee meetings and transcription.

IRB coordinator: will work closely with the CRC to make sure that all IRB applications are up to date

Organizational chart

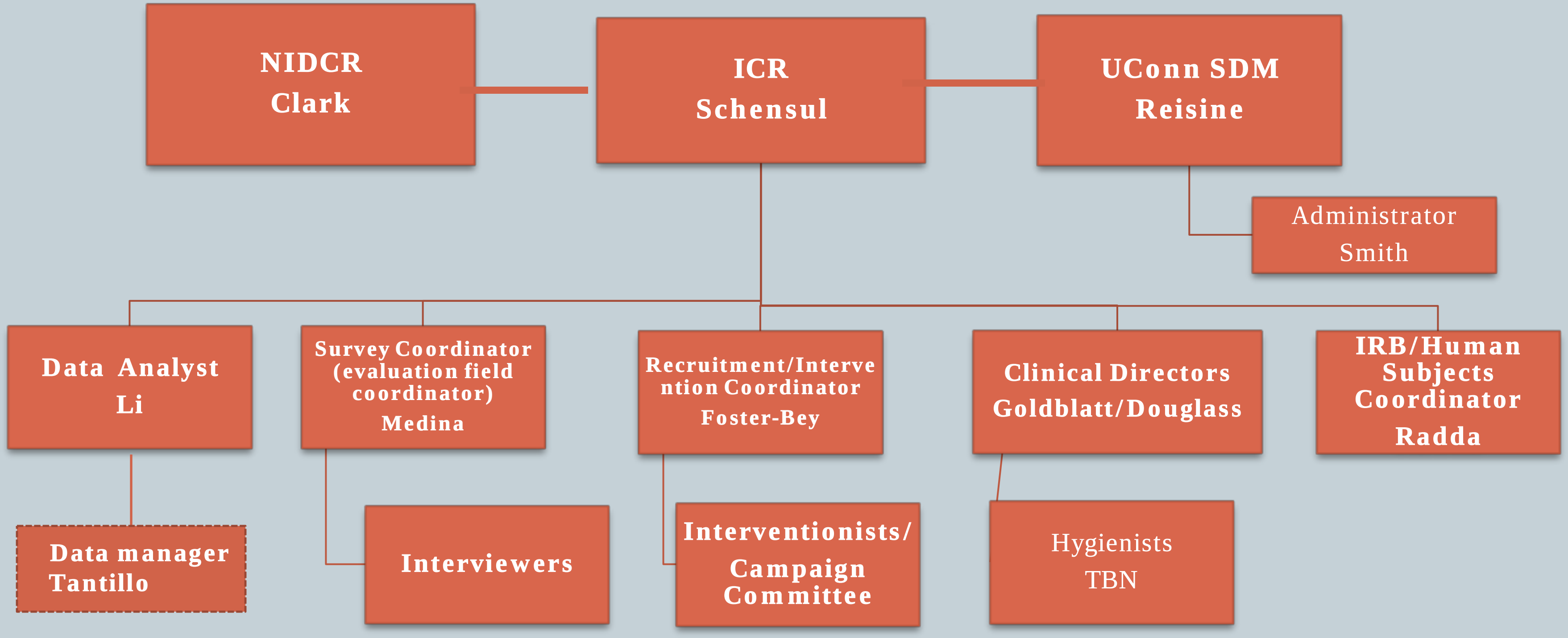

Investigator Responsibilities

- Good Clinical Practice

- Records Retention
  - All source documents will be kept in or transferred into electronic files
  - Data files will be cleaned, de-identified and password protected to be stored on ICR server, UCHC server, and at federal record center for 7 years after the grant is officially closed

Type of Study

- The study is a community effectiveness trial
- It is designed to disaggregate and test the components of an effective intervention against each other, and to compare their cumulative effect
- It is community-based, meaning that it involves the administration of the housing units in helping to coordinate the interventions on site, and members of the affected community in active involvement in the administration of one of the two interventions (CBPR).
- It is rigorous in its assessment of intervention acceptability and fidelity of implementation at both levels.

Study Objectives

Primary Objectives:

1. To assess the separate and combined effectiveness of two components of a cognitive/behavioral intervention that together improve oral hygiene behaviors and oral hygiene status;

2. To assess whether differential sequenced combinations of these components has a differential effect on behavioral and clinical outcomes;

3. To investigate the mediation effects of cognitive/behavioral factors in changing clinical outcomes.

Primary Outcome Measures: Plaque Scores and Gingival Index

Secondary Outcome Measure: Oral Health Related Quality of Life

Sample and Enrollment goals

- Sample size: 450 (from six buildings 75 per building)
- Gender: Males and Females
- Age: Children and adults aged 18-61 with disabilities and those aged 62 and older
- Demographic group: Vulnerable adults or children who are residents of low income senior housing in Central Connecticut. Residents in these buildings must meet income guidelines and be over the age of 62 or be disabled adults or children.
- General health status: The participants in low income senior housing must be able to live independently
- Geographic location: Central Connecticut

Bi-level FFD Design---Matched Building Pairs

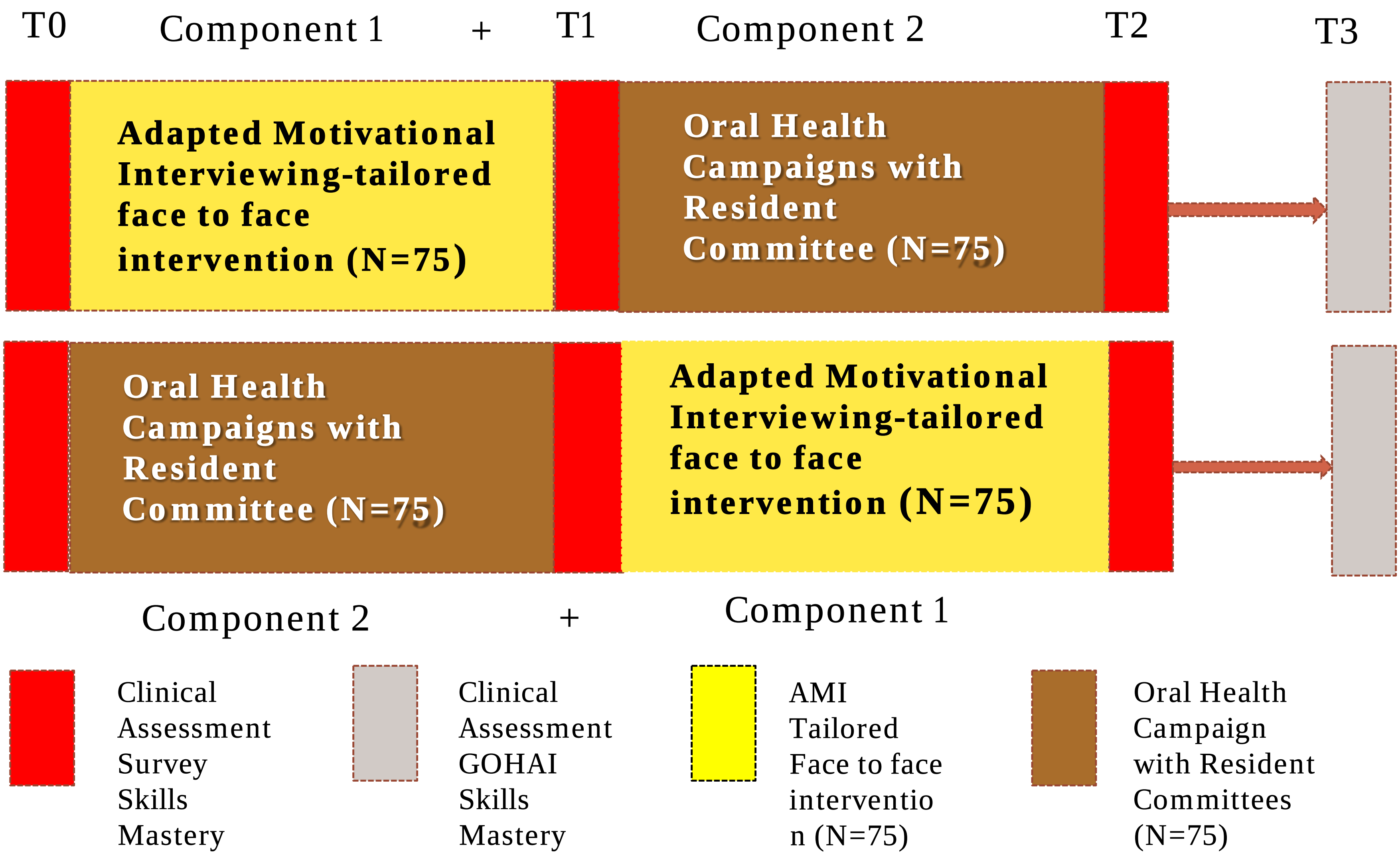

Theoretical Framework

(From Fishbein 2008 and Bandura 1987)

Background Moderators

- Demographic
- Health status
- ADLS*
- CES-D
- Self-assessed oral health status

Intervention (fidelity)

- AMI focal points addressed
- P-M Prepost score
- Exposure to campaign messages (dosage)
- Recognition of campaign messages

Cognitive Behavioral Mediators

- Oral Health Knowledge*
- Perceived Oral health risks*
- OH health consequences*
- OH social support*
- OH self efficacy*
- OH self management fears and worries*
- OH norms/beliefs*
- OH reported behaviors*
- Dry mouth*
- Diet*

Behavioral Intentions

- OH self managemt intentionality *

Outcomes

- Plaque score
- Gingival index
- Behavioral Mastery Score
- GOHAI

* AMI focal points and Campaign messaging

Informed Consent: Forms

Forms include:

- General Study Consent Form (Building AB or BA, as appropriate)
- Consent Comprehension Form
- Authorization to Use and Disclose Protected Health Information for Research Purposes (HIPAA Form)
- HIPAA Authorization to Photograph/Video/Audiotape Form
- Campaign Committee Consent Form

Consent Process

- Consenters are all trained and certified in human subjects protections
- Consenters will use only UCHC IRB stamped forms
- Consent will be explained and administered in English or Spanish, based on language of participant's preference
- All participants will be asked to review and sign the general and 2 HIPPA consent forms.
- Only prospective Campaign Committee members will sign the Campaign Committee Consent Form.
- Consent forms will be kept on file and a signed version of all consent forms will be given to participant
- Confirmation of understanding will be through appropriate response to five questions

Inclusion Criteria

- Disabled children and adults aged 18 years and above, and adults 62 and above, including both male and female building residents, and minorities and non-minorities
- Permanent residence in sample buildings
- Independent of conservator
- Must be able to speak English or Spanish
- Judged competent to participate based on ability to respond correctly to five key questions about information covered during administration of informed consent
- Have two or more natural teeth

Exclusion criteria

- Considered by research staff to be cognitively unable to give informed consent (based on response to five comprehension questions)
- Exhibition of continued disruptive behavior while participating in the project (termination)
- History of infective endocarditis, prosthetic cardiac valve replacement, insertion of an arterial stent in past 6 months, myocardial infarction (heart attack) in past 6 months, joint replacement surgery
- Under conservatorship
- Fewer than two natural teeth

Repeat eligibility criteria (T1-T3): Exclusions

- History of infective endocarditis
- History of prosthetic cardiac valve replacement
- Insertion of an arterial stent in past 6 months
- A myocardial infarction (heart attack) in past 6 months
- History of joint replacement surgery
- Fewer than two natural teeth
- Under conservatorship

Health Status Screener

GOH Eligibility Form

If participant answers yes to any of the following then he/she is **not eligible** for the clinical exam portion of this intervention and, therefore, is **not eligible** to participate in the study. He/she is welcome to participate in the oral health fairs.

T0 ______ T1______ T2______ T3______

1. Have you ever had surgery to replace a joint, such as a hip, knee, shoulder or elbow? (pins, plates and screws do not count)	yes	no
2. Do you have a heart valve replacement?	yes	no
3. Have you had an infection inside your heart? (this means probably had to have antibiotics for 4-6 weeks during your heart infection)	yes	no
4. Have you had a stent (a small tube that keeps your arteries open) placed in your heart within the past 6 months?	yes	no
5. Have you had a heart attack (known as an MI) in the past 6 months?	yes	no
6. Do you have at least two natural teeth?	yes	no
7. Do you have a conservator? (a guardian appointed by a judge to protect and manage your financial affairs and/or your daily life)	yes	no

Study Visit/Schedule of Events

Building A – Tailored intervention first followed by campaign

Visit	Activity	Timing	Compensation
1.– T0	Clinical assessment, skills assessment – 15 minutes	Entry to study	$15.00
2. – T0	Survey – 1 hour	Entry to study	$15.00
3.	Tailored educational session 45 min – 1 hour	1-2 months after entry	None
4. – T1	Survey – 1 hour	1-2 months after educational session	$15.00
5. – T1	Clinical assessment, skills assessment – 15 minutes	1-2 months after educational session	$15.00
	3 Campaign Events/Oral health fairs – 2-3 hours each	2-5 months after T1	None
6. – T2	Survey– 1 hour	1-2 months after campaigns	$15.00
7. – T2	Clinical assessment, skills assessment – 15 minutes	1-2 months after campaigns	$15.00
8. – T3	Clinical assessment, skills assessment, GOHAI – 15 minutes	6-7 months after T2	$15.00

Study Visit/Schedule of Events

Building B – Campaigns first followed by tailored intervention first

Visit	Activity	Timing	Compensation
1.– T0	Clinical assessment, skills assessment – 15 minutes	Entry to study	$15.00
2. – T0	Survey – 1 hour	Entry to study	$15.00
	3 Campaign Events/Oral health fairs – 2-3 hours total	2-5 months after study entry	None
3. – T1	Survey– 1 hour	1- 2 months after campaigns completed	$15.00
4. – T1	Clinical assessment , skills assessment – 15 minutes	1- 2 months after campaigns completed	$15.00
5.	Tailored educational session 45 min. to 1 hour	2-4 months after campaigns completed	None
6. – T2	Survey– 1 hour	1-2 months after campaigns	$15.00
7. – T2	Clinical assessment, skills assessment – 15 minutes	1-2 months after campaigns	$15.00
8. – T3	Clinical assessment, skills assessment, GOHAI – 15 minutes	6-7 months after T2	$15.00

Study Procedures

- Clinical assessments to assess Plaque Score and Gingival Index
- Mastery skills assessment
- A survey administered by trained research assistants
- Administration of the AMI-PM
- Recording of the AMI-PM to assess fidelity of the intervention.
- Training sessions for the campaign committee to develop campaign messages, materials and activities that are consistent with the conceptual model. Examples of materials will be provided to committee members based on the pilot study.
- Three campaign events

Clinical Measurements – Gingival Index

- Gingival Index :
  - Root tips and partially erupted teeth excluded
  - Erupted third molars are included
- Score Criteria

**Appearance**	**Bleeding**	**Inflammation**	**Points**
Normal	No bleeding	None	0
Slight change in color and mild edema with slight change in texture	No bleeding	Mild	1
Redness, hypertrophy, edema and glazing	Bleeding on probing/pressure	Moderate	2
Marked redness, hypertrophy, edema, ulceration	Spontaneous bleeding	Severe	3

Clinical Measurements – Plaque Score

- Plaque Score :
  - Root tips and partially erupted are excluded, erupted third molars are included
  - Present or absent in gingival third of tooth

Training and Calibration Gingival Index and Plaque Score

- Training is divided into three phases as follows:
  - The instructional phase in which examination team members are familiarized with research examination procedures and criteria for research assessments.
  - The standardization phase in which they are trained to use standard procedures and apply standard criteria for the oral health assessments.
  - The calibration phase in which the degree of correlation within and among the examiners and the standard examiner is measured.
  - Retraining and recalibration will be conducted every six months to ensure assessment consistency

Training and Calibration
Oral Hygiene Skills Assessment

- Training is divided into three phases :
  - The instructional phase in which intervention team members are familiarized with research protocol
  - The standardization phase in which they are trained to use standard assessment and evaluation or oral hygiene techniques ☐
  - The calibration phase in which the degree of correlation within and among the examiners and the standard examiner is measured.

Calibration

- The Gold Standard and all interventionists in training will each evaluate 4 patients
- The Gold Standard monitors the calibration session without discussing observations with any of the interventionists or the assistant clinical director.
- Data from the calibration sessions are analyzed to measure correlation within and between each interventionist and the Gold Standard.
- If correlations between each of the interventionists and the Gold Standard are not within acceptable ranges, additional training sessions will be scheduled.

Safety

- Safety: Definitions, Collection, and Reporting
  - Adverse Events (AEs)
  - Serious AEs (SAEs)
  - Unanticipated Problems (UPs)
- Queries resulting from the above

Site File Review/Regulatory Binder

- Investigator Site File Review
- Structure of the Regulatory Binder as well as Essential Documents to include:
  - 1572, 1571, Form 1195 (as applicable)
  - IRB approval documents: protocol, patient handouts, advertisements, consent document
- Document updates

Clinical Monitoring

- Clinical Monitoring
  - Contacts
  - Responsibilities of
  - Frequency
- Close out procedures

ICR Program, Day 2

- Study process overview
- Participant walk through
- Data collection/source documents
- Investigator tour of facilities and study site
- Site file review
- Review and action steps

Participant Walkthrough

- Recruitment and Initial Screening process

  Enrollment and Assessments

- Intervention
- Using the Data Tracking System for implementation

Clinical Armamentarium and Assessment

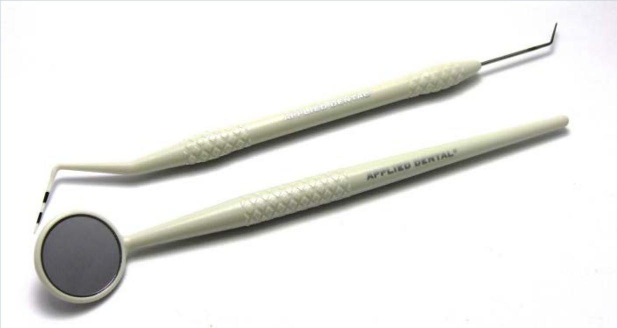

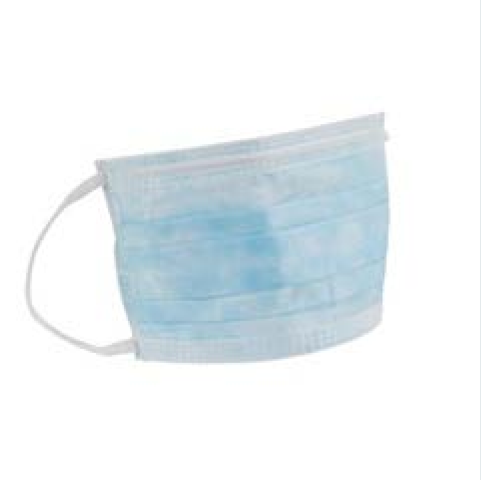

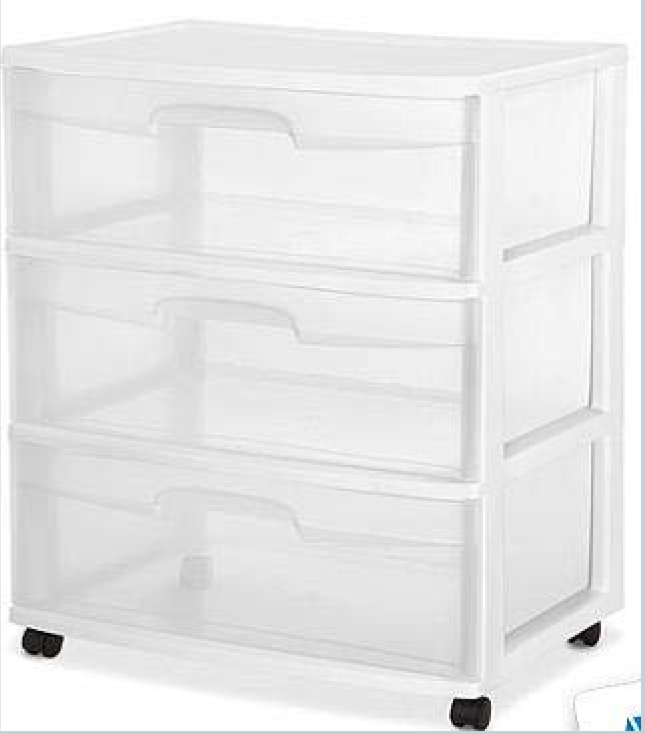

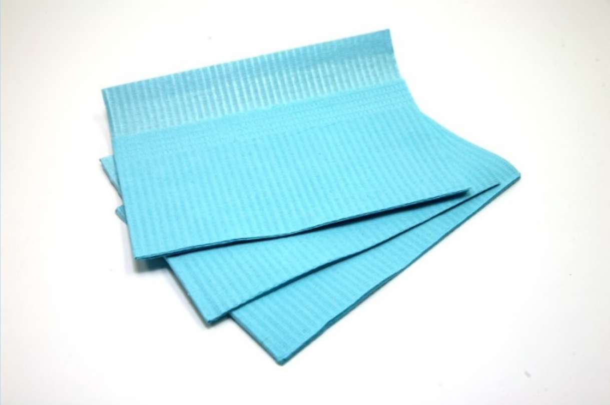

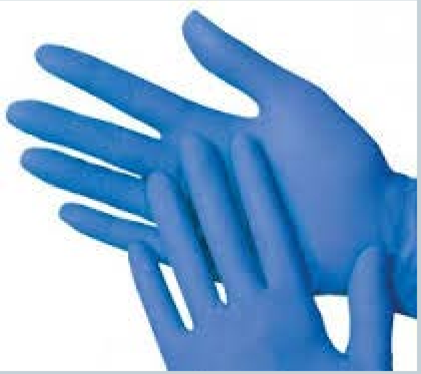

Recruitment and Screening

- Participants will be recruited through bilingual presentations (2), fliers and individual conversations.
- Participants will indicate interest on signup sheets at events.
- Non-attendees and those not screened at the time of the presentations will be followed up through other events, and study team regular presence in the buildings.
- Participants will be screened in privacy in English or Spanish at signup events to determine initial eligibility.
- Eligible participants will be given a date and time for consenting, clinical assessment and mastery assessment on appointment cards. Appointment will be registered on a group calendar.
- Date and time will be entered into tracking data base on site.

Re-screening, Consent, Enrollment and Assessment

- At appointment participants will be administered repeat eligibility screening
- Consent administration
  - Explanation in English or Spanish
  - Reading of consent form with explanation
  - Five questions for verification of understanding
- Signature and copies of all consent forms for file and for participant
- Those who consent successfully will be administered the Mastery Assessment by research staff
- For Committee Members:
  - Consent read and signed at first meeting
- Clinical assessment explained and conducted
- Participants will be entered into the tracking data base.
- Appointment for the survey in Spanish or English.
- Scheduled appointments will be entered into the tracking data base

Survey Administration: Preparation

- Receive interview schedule and master ID.
- Interview schedule produced from Access queries
- Survey packets in files with participant ID in locked cabinet in Zahira’s office
- Survey packets include:

  1 Packet Checklist (content of package)
  1 Participant Record Checklist
  1 Signed Consent forms (Eng or Sp) –
  1 audio consent form
  2 Signed HIPAA forms (Eng or Sp)
  Completed Eligibility Form
  Re-Eligibility Form-(blank)
  1 campaign consent form
  1 UCHC Consent Addenda (Eng or Sp)
  1 Payment envelope ($15.00)

- Reminders until confirmation
- Interviews are conducted in private location
- After completion participant is paid $15.00. Receipts to participant and in file

Interventions

AMI

CAMPAIGN

AMI - Preparation

- A completed custom-constructed focal point checklist.
- Copy of baseline clinical assessment results
- Oral Hygiene Skill Assessment Evaluation Form
- Copy of blank Audio consent form
- Practice to Mastery equipment: mouth models, tooth/denture brushes, floss
- Laptop loaded with: educational videos for instructions on proper brushing, flossing, and denture care; results of the baseline skills assessment; a blank form for Personal GOH Plan
- Audio recorder
- Portable printer, paper, and extension cord
- Handouts with instructions on proper brushing, flossing and denture care that reflect the instructions in the educational videos
- Oral hygiene kit (toothbrush, toothpaste, denture brush, tongue cleaner, floss, and floss handle

Cutoff points linking survey to focal point checklist

Domain	Cut-off points
1. ADLs	Need help on any of these
2. Oral health knowledge	<5 correct
3. Oral health self-efficacy (a)	Mean of items <3 (disagree and strongly disagree)
3. Locus of control (b)	If response to this item was agree or strongly agree)
4. Oral Health Norms - Beliefs about Importance of oral hygiene	1 or 2 on any item (Not at all important; not very important)
5. Oral health Social Support -	If one or more items is “0” (none)
6. Oral hygiene behaviors	<2 times per day
7. Perceived Oral Health Risks	Mean >=3 (1, very unlikely; 2, very likely; 3, likely, 4 very likely)
8. Self-management worries	Mean <3 for scale (4 = not at all; 3= not much)
9. Self-management fears	Mean < 3 (4= not at all; 3 = not much)
10. Oral Health Self-Management Intentionality	Mean <1 (0= no possibility; 1= slight possibility)
11. Dry mouth	Yes
12. Diet	>2-3 times a day on any item.
Clinical Assessment	All participants

Focal Point Checklist

Check list - Focal Points		
**Focal Point**	√	**Describe problem**
**Intervention Domain 1:** ADLs/IADLs		
**Intervention Domain 2:** Knowledge		
**Intervention Domain 3:** Self-efficacy/locus of control		
**Intervention Domain 4:** Oral Health Beliefs		
**Intervention Domain 5:** Social Support		
**Intervention Domain 6:** Oral hygiene behaviors		
**Intervention Domain 7:** Perceived oral health risk		
**Intervention Domain 8:** Self-Management Worries		
**Intervention Domain 9:** Self-Management Fears		
**Intervention Domain 10:** Oral health self-management intentionality		
**Intervention Domain 11:** Dry Mouth		
**Intervention Domain 12:** Diet		
**Review of Clinical Assessment**		
o Gingival Levels		
o Plaque Score		

AMI-Practice to Mastery Skills Development Process

- Discuss with participant the importance of taking care of teeth, mouth, gums in keeping one well and healthy.
- Explain areas of concern based on survey and show the focal point checklist
- Prioritize and discuss these areas of concern and how to remedy them
- Skills development
  - Review results of baseline assessment using typodont
  - Show videos and discuss brushing and flossing.
  - Demonstrate
  - Participant demonstration (maximum 3 times)
  - Complete Mastery assessment.
- Develop personal action plan with participant

Oral Health Assessment Form

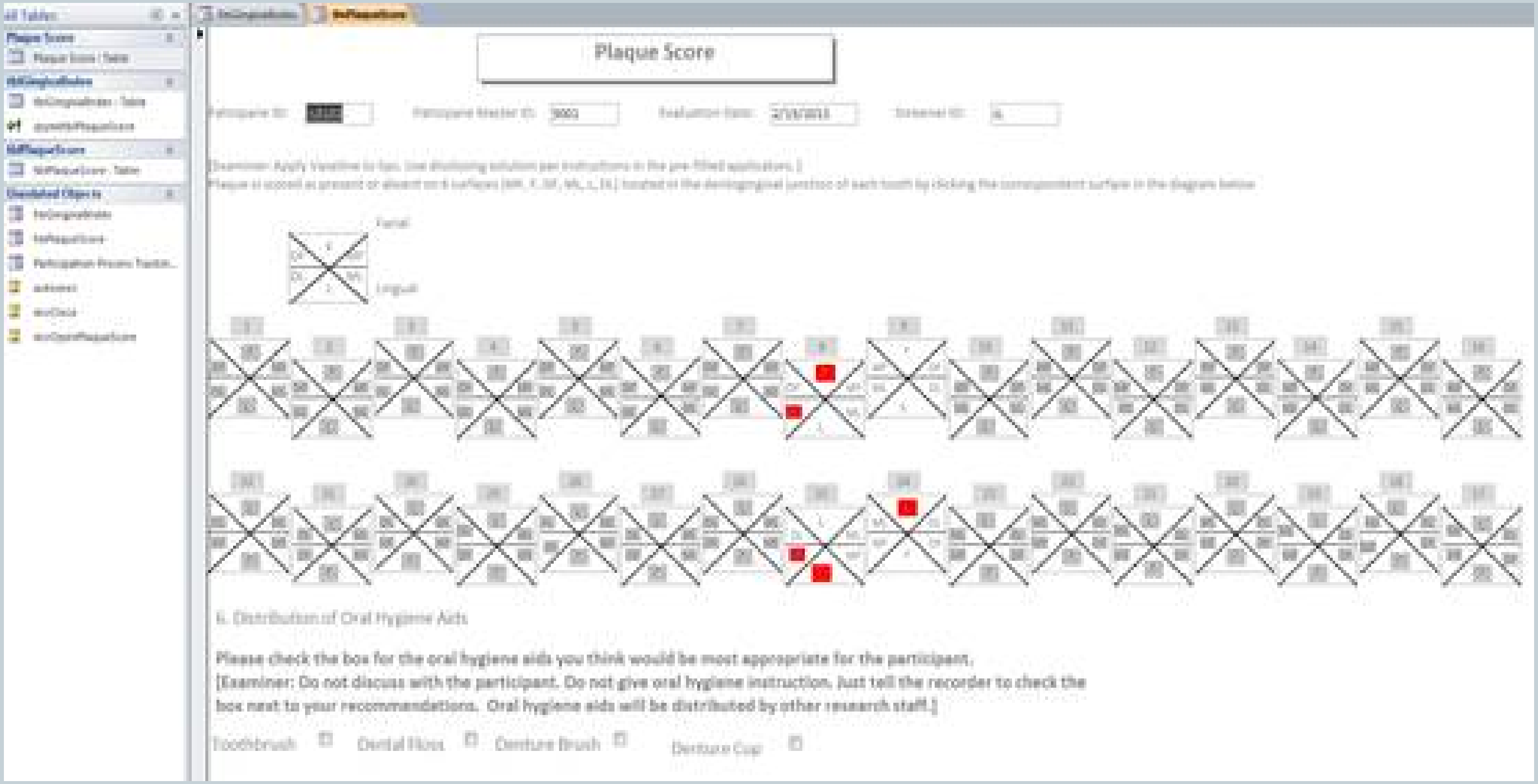

Skills Assessment Form

Behavioral skills assessment form checklist						
Mastery Level						
Skill	Excellent (needs no prompting)	Good (needs no more than 2 corrections)	Fair (requires 3 or more corrections)	Poor (requires complete instruction)	Lacks manual dexterity due to physical disability ie. Arthritis, visual impairment	Sensory impairment limiting or modifying oral hygiene instruction
Tooth brushing						
Flossing						
Cleaning Dentures or Partials						

Plan

- Prepare plan based on intentions of participants.
- All plans should include brushing and flossing properly plus at least one cognitive/behavioral domain (e.g. adjusting for ADL; dealing with worries or fears)
- Copy of plan for file and for participant printed on site
- Participant gets an information packet
- Tracking data entered into tracking form
- Intervention data (elements of plan) entered same day into data base

Personal GOH Plan

Problem Domain	Participant’s Plan to Address Problem
BRUSHING	
FLOSSING	
PHYSICAL PROBLEM<br>CARING FOR YOUR MOUTH	

Data Entry Form - ACCESS

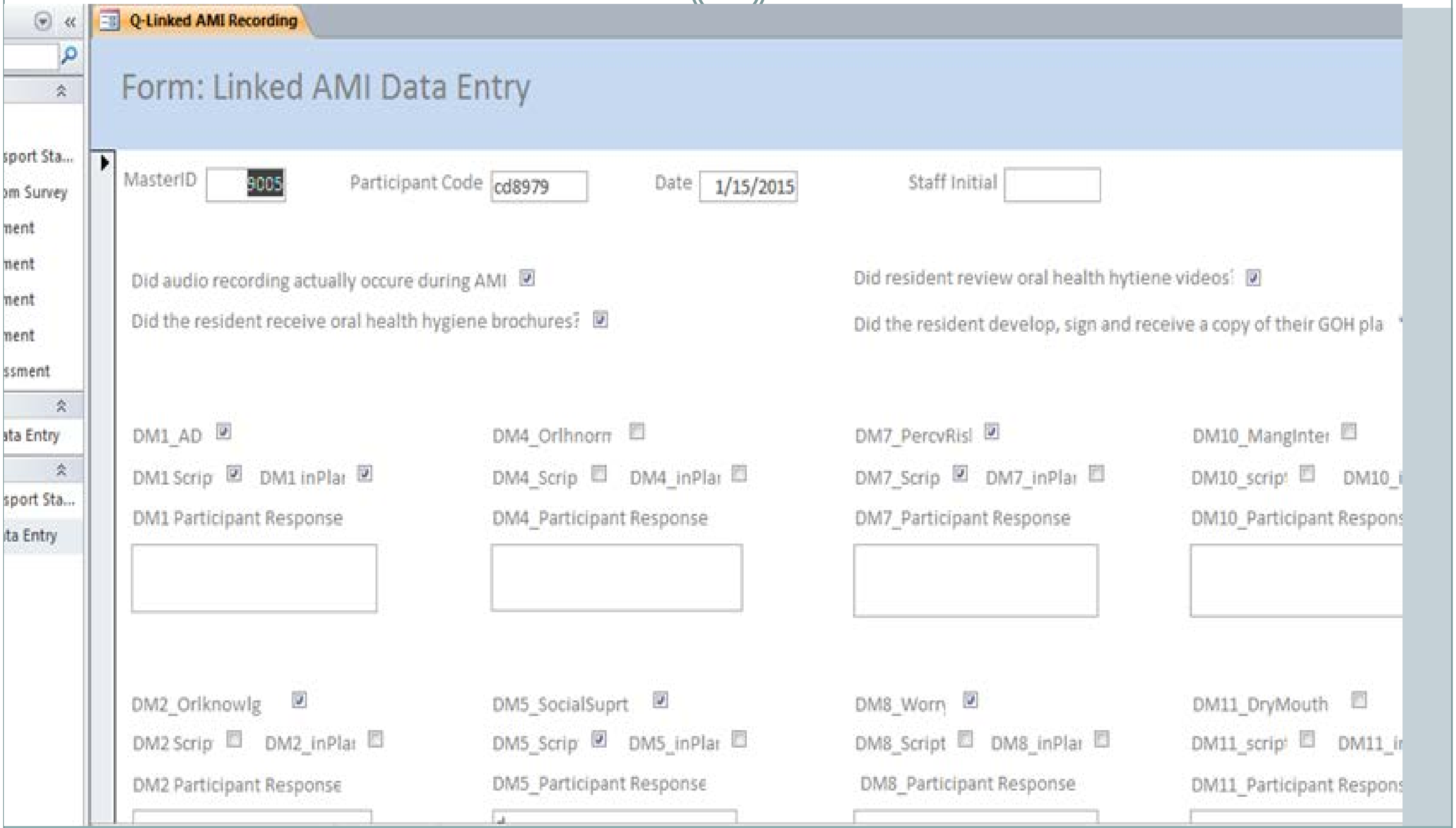

Campaign Planning

- Recruit committee (up to 10 people)
- Train committee (6 – 10 sessions)
  - MODULE 1 - Session 1: Building group identity and scope of work
  - MODULE 2 - Sessions 2 & 3: Review of the components of the Pro-GOH Campaign; Protecting and respecting other residents; Effective communication
  - MODULE 3 - Sessions 4 & 5: Oral health and oral health self-management behavior; Confirmation of campaign event schedule
  - MODULE 4 - Session 6 & 7: Creation of a campaign plan
  - MODULE 5 - Sessions 8 & 9: Development of campaign material
  - MODULE 6 - Session 10: Preparation for campaign events
  - Additional 2 sessions to finalize campaign and practice scripts

Campaign Administration

- Prepare messages, materials and stations
- Ensure location and times for events
- Invite residents and “experts”
- Promote events in building
- Identify staffing and placement of stations
- Prepare process for assigning and collecting passports on entry and exit.

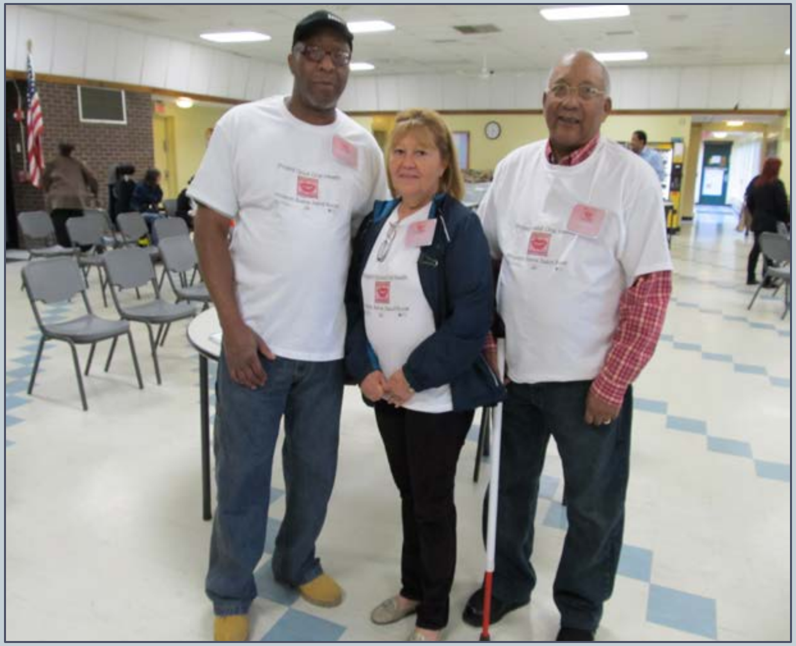

Passport

Source Documents (1)

Outcome Evaluation Assessment	Content	Collection Format	Quality Control
**Survey**	Demographics; general health history & status; oral health history & status; oral health experience and decision-making; reported oral health behavior; Scales measuring the study's theoretical domans 1 – 12; access to oral health care;	QDS	Built-in Control; Immediate survey review; random audit; systematic logic check/cleaning in SPSS
**Clinical Assessment**	Gingival Index and Plaque Scores (open link to CAF) DentalExam (01092015).accdb	Access	Calibration & re-calibration; Built in control Random audit
**Oral Hygiene Skills Assessment**	Mastery of brushing and flossing skills	Access	Built in control; Queries; Random audit
**Campaign Participation/ Dosage**	Attendance and participation at oral health activities assessed through stamped passports indicating participation at specific activities.	Access	Built in control; Queries; Random audit

Source Documents (2)

Intervention Process/Fidelity Documents	Content	Collection Format	Quality Control
**Face to Face AMI Delivery**	Survey export focal points to be addressed<br>Scripted message for each of the 12 domains;<br>Participant comments entered into access data base<br>Intervention Process Tracking (01282015).accdb	Audio record<br>Access	Random audit<br>Observation
**Campaign Committee**	Record of participation at training sessions, content and attendance	Paper	Observation<br>Note taking
**Campaign Participation/ Dosage**	Attendance and participation at oral health activities assessed through stamped passports indicating participation at specific activities.	Access	Built in control;<br>Queries;<br>Random audit
**Other Data**	**Content**	**Collection format**	**Quality Control**
**Participation Tracking Database**	Participants' basic demographics, contact information, scheduling and completion of each steps of evaluation and intervention activities<br>Participant Tracking 02062015.accdb	Access	Multiple queries to compare entries with evaluation, intervention, and field records

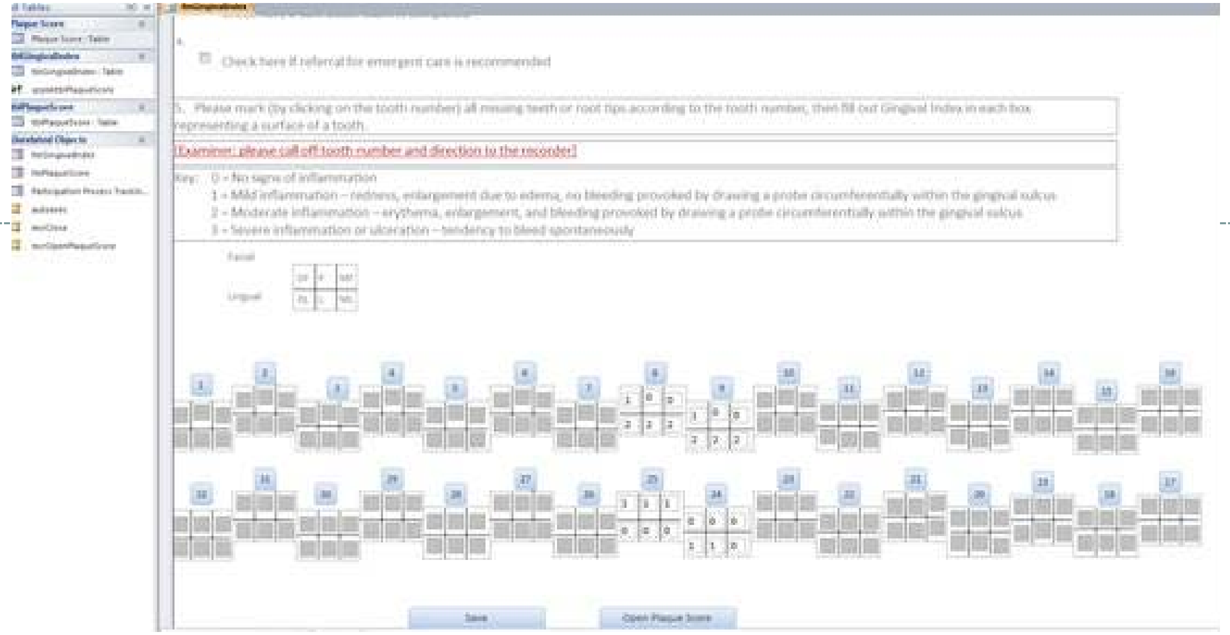
Check here if referral for emergent care is recommended
5. Please mark (by clicking on the tooth number) all missing teeth or root tips according to the tooth number, then fill out Gingival Index in each box representing a surface of a tooth.
[Examiner: please call off tooth number and direction to the recorder]
Key: 0 = No signs of inflammation
1 = Mild inflammation – redness, enlargement due to edema, no bleeding provoked by drawing a probe circumferentially within the gingival sulcus
2 = Moderate inflammation – erythema, enlargement, and bleeding provoked by drawing a probe circumferentially within the gingival sulcus
3 = Severe inflammation or ulceration – tendency to bleed spontaneously
Facial
Lingual
Save
Open Plaque Score

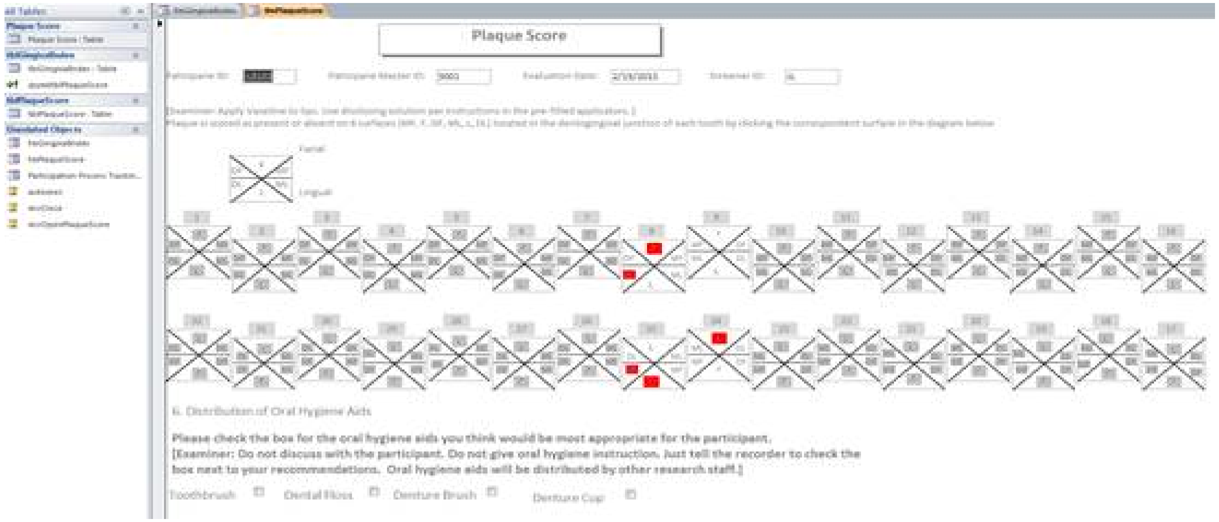
Plaque Score
6. Distribution of Oral Hygiene Aids
Please check the box for the oral hygiene aids you think would be most appropriate for the participant.
[Examiner: Do not discuss with the participant. Do not give oral hygiene instruction. Just tell the recorder to check the box next to your recommendations. Oral hygiene aids will be distributed by other research staff.]
Toothbrush
Dental Floss
Denture Brush
Denture Cup

Record Checklist (for file)

Participant ID #: ___________________

	**Forms Completed & In Folder ("√")**	**Comments**
Study Consent Form		
Consent Comprehension Form		
HIPAA		
Audio/Video/Photo Authorization Form		
Campaign Consent Form		
Focal Points Checklist		
Personal GOH Plan		

Forms Completed and in Folder (put "√")

	**T0**	**TAMI**	**T1**	**T2**	**T3**	**Comments**
Eligibility Screening Form						
Clinical Assessment Form						
Skills Assessment Form						
Oral Health Fair Passports						

Data Documentation and Transfer

DOCUMENTATION
PAPER OR ELECTRONIC DATA CAPTURE (EDC) CRF
DISCUSSION

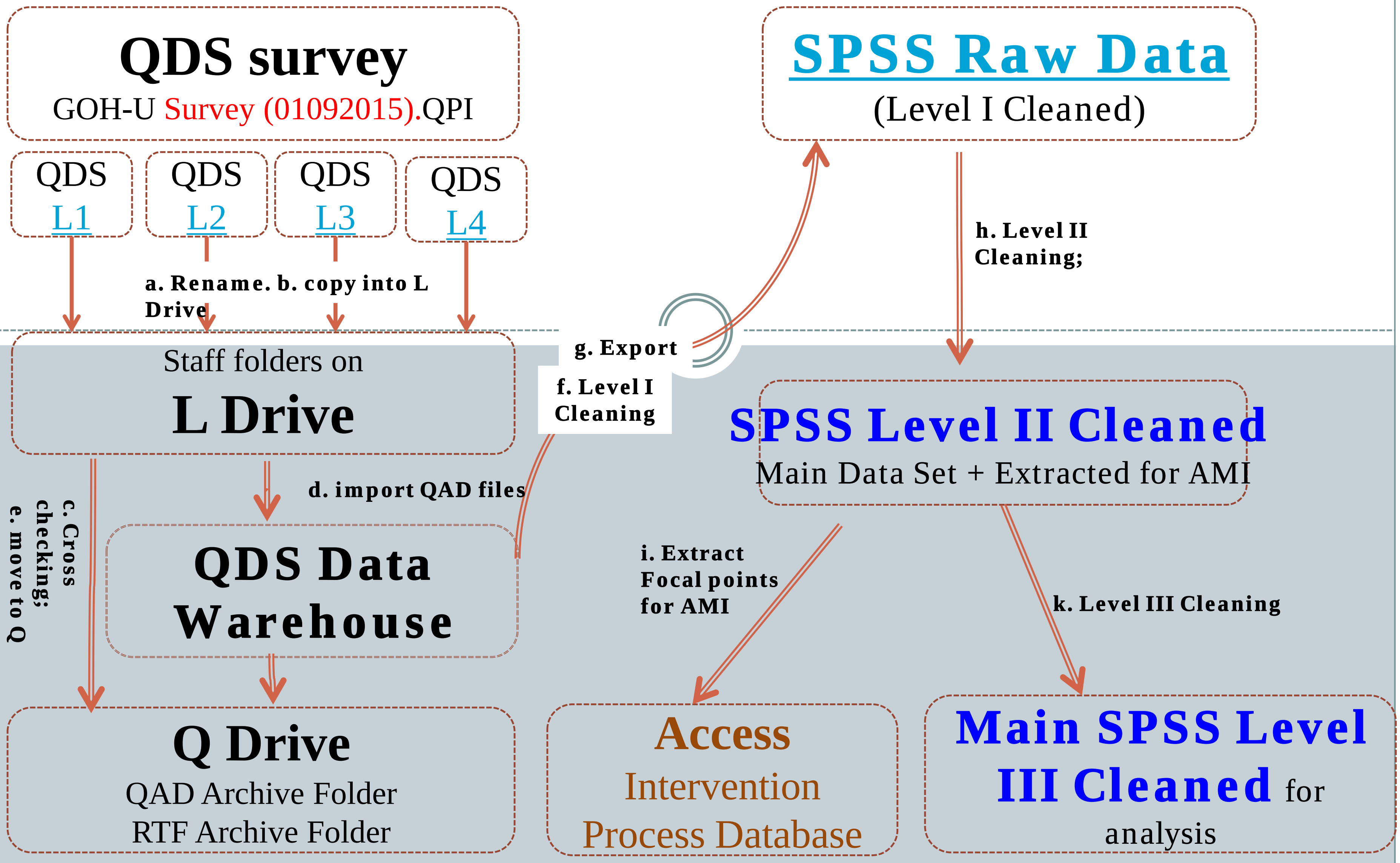

GOH SURVEY DATA FLOWCHART

Investigator Site File Review

- Materials Binder _ TOC_ draft(1) FINAL.docx

Training Log

Staff Training Activity	Initial training date	Print	Signature
Recruitment	1/8/15		
Enrollment and Consent	1/8/15		
Survey Administration	1/13/15		
Mastery Assessment	1/14/15		
Calibration (dental assessment)	1/14/15		
Calibration (mastery assessment)	1/14/15		
Using the Tracking Database	1/9/15; 1/12/15; 1/13/15; 1/21/15		
AMI – PM Process	1/15/15		
Campaign Training and administration	1/20/15		
