# Supplementary material for: Improving Oral Health in Older Adults and People With Disabilities: Protocol for a Community-Based Clinical Trial (Good Oral Health)
Source: JMIR Res Protoc. 2019 Dec 18;8(12):e14555. doi: 10.2196/14555 (PMC6939248; doi:10.2196/14555)
Supplement: Multimedia Appendix 2 [file resprot_v8i12e14555_app2.pdf]

SUMMARY STATEMENT
(Privileged Communication)

PROGRAM CONTACT:
Melissa Riddle
riddleme@mail.nih.gov

Release Date: **12/11/2013**

Application Number: **1 U01 DE024168-01**

Principal Investigators (Listed Alphabetically):
REISINE, SUSAN T PHD (Contact)
SCHENSUL, JEAN J. PHD

Applicant Organization: UNIVERSITY OF CONNECTICUT SCH OF MED/DNT

Review Group: **ZDE1 VH (22)**
National Institute of Dental and Craniofacial Research Special Emphasis Panel Clinical Trial or Biomarker Clinical Evaluation Study Planning Grant and Clinical Trial Implementation or Biomarker Clinical Evaluation Study Cooperative Agreement Applications Review Panel

Meeting Date: **11/06/2013** ***RFA/PA:*** **PAR11-339**
Council: **JAN 2014** ***PCC:*** **Q2A**
Requested Start: **04/01/2014**

Project Title: **Good Oral Health: a Bi-level Intervention to Improve Older Adult Oral Health**

SRG Action: **Impact Score: 22**
Next Steps: **Visit http://grants.nih.gov/grants/next_steps.htm**
Human Subjects: 30-Human subjects involved - Certified, no SRG concerns
Animal Subjects: 10-No live vertebrate animals involved for competing appl.
Gender: **1A-Both genders, scientifically acceptable**
Minority: **1A-Minorities and non-minorities, scientifically acceptable**
Children: **1A-Both Children and Adults, scientifically acceptable**
NIH Defined Phase III Clinical trial

Project Year	Direct Costs Requested	Estimated Total Cost
1	499,560	841,453
2	499,933	842,081
3	499,550	841,436
4	498,403	839,504
5	497,096	837,302
TOTAL	2,494,542	4,201,775

ADMINISTRATIVE BUDGET NOTE: The budget shown is the requested budget and has not been adjusted to reflect any recommendations made by reviewers. If an award is planned, the costs will be calculated by Institute grants management staff based on the recommendations outlined below in the COMMITTEE BUDGET RECOMMENDATIONS section.

1U01DE024168-01 REISINE, SUSAN T (contact); SCHENSUL, JEAN J

SCIENTIFIC REVIEW OFFICER'S NOTES

RESUME AND SUMMARY OF DISCUSSION: The proposed U01 clinical trial is designed to address disparities in oral health in two vulnerable populations with accumulated oral disease: low-income, primarily minority older adults as well as persons with disabilities. The proposal addresses a significant public health problem and if successful, the results of the study will likely have a high impact in the field, both from a methodological as well as an outcome perspective. The intervention project is unique in that it integrates peer education, activities, plus individual preventive behavior to improve oral health, in the locations where they reside. This approach is unique in its multi-level focus on the individual and community and the factorial design is innovative. The principal investigator and multidisciplinary study team are strong and complement each other. The team has produced solid foundational work to support the proposed clinical trial. The Manual of Procedures (MOP) is well developed which suggests that they will be able to initiate the study without much delay. The well-considered design consists of two interventions to increase prevention within these populations. The two approaches are (1) Adapted Motivational Interviewing combined with Practice-to-Mastery (AMI-PM) and (2) a targeted building-level campaign that includes principles of practice to master (CA-PM). The intervention will be presented in differing orders and measurement will compare the short and long term effects. This design should be able to identify the contributions of the various components of the proposed intervention although a concern was raised involving the justification and appropriateness of having two primary outcome measures (plaque index and gingival index). It is unclear if the two primary outcome measures will be somehow combined into a single outcome or if there will be any adjustments for the total number of teeth per subject. There is a concern about the sample population regarding the total number of people in each building from which they are being sampled and recruited. It is unclear whether there is an adequate population at each building from which to recruit or conversely, if there is a plan to accommodate additional subjects that want to participate at a given building. While the study sample size may be sufficient; given this population, it is not clear if the given estimates of patient attrition are appropriate. The plans for patient recruitment and retention are well outlined and a clear data safety monitoring plan is provided. Although the protections of human subjects plan is adequate, given the literacy levels of the subjects and potential competency issues for residents under receivership, additional protections may be necessary. Overall, these concerns do reduce the high enthusiasm for this outstanding application to address oral health disparities. If successful, this study could develop an intervention that may provide a model program to reduce oral health disparities within Connecticut and nationally.

DESCRIPTION (provided by applicant): This application will extend a successful NIDCR-funded bi-level oral health self-management pilot conducted among older/disabled adults residing in one older adult residential building to six buildings and evaluate it using a modified fractional factorial design. The theoretical framework is based on Fishbein's modified theory of reasoned action called the Integrated Model (IM) of Behavioral Prediction. It is operationalized through Adapted Motivational Interviewing, an interactive tailored cognitive approach, and Practice-to-Mastery (AMI-PM). The intervention includes two components: 1) a face to face administration of the AMI-PM; and 2) a targeted building-level campaign that includes principles of practice to master (CA-PM). The specific aims are to: 1) Test the two main components of a bi-level intervention to improve clinical oral hygiene outcomes in relation to one another, and through differential sequencing. 2) Identify cognitive and behavioral mechanisms contributing to clinical oral hygiene outcomes. 3) Assess impact and sustainability of behavioral and clinical outcomes over time. The intervention will be carried out in three cycles of 150 participants each for a total of N=450. Each cycle will include two buildings matched by size and ethnic/linguistic composition housing older adults and those with disabilities. In one condition, the individual level intervention will be introduced followed by the group level intervention. In the second condition, the group level intervention will precede the individual level. Survey and clinical measures taken at

baseline, and following the administration of the first and second components. This design will enable comparison of the short term efficacy of the individual versus group level component (T1 - T2) and the shorter and longer term efficacy of the interventions (T3 - 3 months). Clinical measures at T4, 6 months later, will evaluate sustainability of effect. Primary outcome measures are gingival index and plaque score, both of which improved significantly in the pilot study. Significance lies in the potential of the intervention to address critical disparities in oral health through oral health promotion initiatives in the locations where they reside as well as the ability to evaluate the intervention efficiently using a design alternative to the RCT.

PUBLIC HEALTH RELEVANCE: The proposed study's primary significance lies in the potential of this bi-level intervention to address significant disparities in oral health and consequent morbidity or reduced quality of life among vulnerable, low-income and primarily minority older adults and those with disabilities through prevention in the locations where they reside. The intervention could provide a model for programs to reduce oral health disparities across Connecticut and nationally.

CRITIQUE 1:

Significance: 1
Investigator(s): 2
Innovation: 2
Approach: 2
Environment: 1

Overall Impact: This project seeks to identify an effective intervention to change oral health behaviors and behavioral mediators in residents of senior housing apartments. The approach is unique in its multi-level focus on the individual and community and the factorial design is innovative. The PI and team are strong and complement each other. The foundational work is solid. This application addresses a significant public health problem and the results will likely have a high impact in the field, both from a methodological as well as an outcome perspective.

1. Significance:
Strengths
- The application addresses a problem of public health significance that is understudied.
- The results are likely to be highly replicable and generalizable given the subject recruitment projected.
- The approach to the methodology and design of the trial will inform the literature and impact the design and procedure of other public health trials in the future.
- The focus on an ethnically diverse population is a strength.

Weaknesses
- *[Not Addressed by Reviewer]*

2. Investigator(s):
Strengths
- The investigative team is highly qualified and compliments each other. There is expertise in behavioral medicine, behavioral theory, dental medicine, community outreach and intervention and statistical analysis.

Weaknesses
- There does not appear to be any evidence that any of the team members have published previously together although they appear to have worked together in the past.

3. Innovation:

Strengths
- The decision to NOT do an RCT is innovative and will inform the literature moving forward.
- While most of the measures appear to be standard, the measure of dental fear is innovative.

Weaknesses
- *[Not Addressed by Reviewer]*

4. Approach:
Strengths
- The study design – a focus on individual and community intervention is a significant strength
- The investigators have carefully and adequately addressed intervention fidelity and training of the measurement and intervention staff.
- The measures proposed have been validated and tested previously in this population group.
- The dose of the intervention will be carefully measured and the behavioral framework of the mediators and moderators has been well articulated.

Weaknesses
- It is not clear why the design includes the question of order of the intervention components. Nor is it clear why the investigators hypothesized why order of one over the other could influence outcome measures.
- Attrition of 15% was based on pilot data from what may have been a shorter intervention. It's reasonable to assume that attrition could be higher in a longer trial although the length of the pilot study is not clear.
- Finally, it was unclear what the length of time between T1, T2, and T3 was. Will there be an effort to manage when subjects will be assessed based on when they entered the trial as the assessment windows appear to be quite wide.

5. Environment:
Strengths
- The environment at both institutions is strong and there is significant support from the apartment complexes where subjects will be recruited.

Weaknesses
- *[Not Addressed by Reviewer]*

<u>Clinical Trial Documentation</u> (Study protocol, Clinical Investigator's Brochure or equivalent, and MOP or equivalent):
- The study protocol appears to be complete and is appropriately detailed to allow for replication and fidelity of the intervention components. Measurement tasks are well-defined and quality control procedures and staff training has been well documented.

<u>Plans for Patient Recruitment/Retention</u>:
- Patient recruitment and retention efforts have been outlined and are sound. This research group has established a good track record for recruitment and retention of participants in the past.

<u>Safety Monitoring</u>:
- The investigators propose to use a data safety monitoring board consisting of 3-5 experts with no relationship to the study. Their plans for reviewing, documenting and reporting appears to be consistent with maintaining subject safety in this very low risk trial.

Protections for Human Subjects: Acceptable Risks and/or Adequate Protections
Data and Safety Monitoring Plan: Acceptable
  - The study plans to use a DSMB and the investigators have outlined necessary procedures for managing adverse events and other study related risks.

Inclusion of Women, Minorities and Children:

G1A - Both Genders, Acceptable
M1A - Minority and Non-minority, Acceptable
C1A - Children and Adults, Acceptable

- Both genders will be recruited (40% male) and 62% of the sample will be African American with 40% being Hispanic. Children between the ages of 18-21 will be included but they will likely make up less than 2% of enrollment.

Vertebrate Animals: Not Applicable (No Vertebrate Animals)

Biohazards: Not Applicable (No Biohazards)

Budget and Period of Support: Recommend as Requested

CRITIQUE 2:

Significance:	2
Investigator(s):	3
Innovation:	2
Approach:	3
Environment:	2

Overall Impact: This application is to develop and test approaches to improve oral health among older/disabled adults residing in older adult residential buildings. It consists of two interventions to increase prevention within these populations. The two approaches are 1) Adapted Motivational Interviewing combined with Practice-to-Mastery (AMI-PM) and 2) a targeted building-level campaign that includes principles of practice to master (CA-PM). The intervention will be presented in differing orders and measurement will compare the short and long term effects. This is a significant research topic with the potential to have an important impact on an expanding part of the population. If successful it is also likely to reduce the disparities present in this group. The study team has clearly made good use of their R34 funding. They have proposed a well-considered design that should identify the contributions of the various components of their proposed intervention. The MOP is well developed which suggests that they will be able to initiate the study shortly after funding is received. While the study sample seems sufficient it is unclear whether there is a adequate population from which to recruit. Reviewers will consider each of the first five review criteria below in the determination of scientific and technical merit, and give a separate criterion score for each. Subsequent review criteria are factored into the Overall Impact score but do not receive an individual criterion score.

1. Significance:
Strengths

- Addresses an important problem; the oral health of community-residing low income, disabled and minority adults.
- Tests possible approaches to improve oral health in specified population based on approaches developed in prior R34
- Supported by theory and preliminary data
- Personalized intervention
- Involves study subjects in the development of part of the interventions
- Well formulated MOP

Weaknesses

- None

2. Investigator(s):

Strengths

- Study team is well suited to conduct study
- PIs complement each other
- Statistician is qualified to conduct suggested analysis
- R34 has provided prior experience resulting in a strong team

Weaknesses

- It would have been nice to have included a hygienist as a co-investigators, since the intervention has major hygiene components

3. Innovation:

Strengths

- Uses existing theoretical concepts but in an innovative approach to improving the oral health of community-residing low income, disabled and minority adults
- The use of a modified fractional factorial design (MFFD) is an important and innovative approach in dealing with some of the size and expense issues found in RCTs
- The use of a multilevel intervention with a design that allows disentanglement of its various components

Weaknesses

- None

4. Approach:

Strengths

- Used the R34 to develop a comprehensive MOP
- Overall strategy to test hypotheses is appropriate
- Plans to track study fidelity are present
- Clearly stated study variables
- Study hypothesis are clearly stated and testable
- Protection of human subjects is adequate

Weaknesses

- How will they handle it if more study subjects wish to participate than planned
- No plan if recruitment falls short
- It is not stated what the overall size of the population from which recruitment will occur and what the expected response rate is
- As buildings are already chosen the numbers of potential study subjects should be known but was not mentioned

5. Environment:

Strengths

- They already have commitments from residences
- The scientific environment is well suited to support this study

Weaknesses

- none

Clinical Trial Documentation (Study protocol, Clinical Investigator's Brochure or equivalent, and MOP or equivalent):

- The MOP is well developed

Plans for Patient Recruitment/Retention:

- Lacks indication of the number of study subjects available for recruitment

Safety Monitoring:

- Satisfactory

Protections for Human Subjects: Acceptable Risks and/or Adequate Protections
Data and Safety Monitoring Plan: Acceptable

Inclusion of Women, Minorities and Children:
G1A - Both Genders, Acceptable
M1A - Minority and Non-minority, Acceptable
C1A - Children and Adults, Acceptable

Vertebrate Animals: Not Applicable (No Vertebrate Animals)

Biohazards: Not Applicable (No Biohazards)

Resource Sharing Plans: Acceptable

Budget and Period of Support: Recommend as Requested

CRITIQUE 3:

Significance:	2
Investigator(s):	1
Innovation:	2
Approach:	3
Environment:	1

Overall Impact: The proposed five year project has many Strengths, and is a broad-based approach to promoting oral health self management in older and/or disabled adults living in a large urban housing buildings. Bi-level interventions (i.e., group-based and individually-tailored) are planned with predominantly ethnic/racial minority older adults and disabled individuals. Both these groups suffer from a disproportionate burden of oral disease. The project itself, and the proposed interventions, are theory-based. While the project overall is has considerable merit, and is highly significant from a public health perspective, certain methodological questions and issues dampen enthusiasm.
Reviewers will consider each of the first five review criteria below in the determination of scientific and technical merit, and give a separate criterion score for each. Subsequent review criteria are factored into the Overall Impact score but do not receive an individual criterion score.

1. Significance:
Strengths
- There are significant disparities in oral health that disproportionately affect older adults, particularly ethnic/racial minorities, and individuals with disabilities. These groups then are vulnerable to oral and systemically-related diseases, and to decreased oral health-related quality of life.
- With the focus on entire housing units, there is potential for considerable simultaneous impact on the oral health status of hundreds of ethnic/racial minority and other older adults, as well as individuals with disabilities.

Weaknesses
- The generalization beyond urban housing units for older adults and disabled individuals is questionable, although there may be implications for similar group housing situations.
- The sustainability of the program will require its adoption by the administration of the housing units, social service agencies, health care delivery entities, and/or components of local government. -

2. Investigator(s):

Strengths

- This multiple PI (n = 2) project includes an investigational team that is well prepared to conduct the proposed research.
- The Institute for Community Research (ICR) will partner with the University of Connecticut School of Dental Medicine in all aspects of the conceptualization and conduct of the proposed study, as well as data collection, analysis and publications. In addition to sharing guidance and direction of the study ICR will take primary responsibility for field operations including recruitment, intervention administration, and survey and practice-to-mastery data collection, data management, and preparation of data for data sharing. ICR will work collaboratively with the School of Dental Medicine to ensure effective collection of oral health screening data, coordination of SDM resources in the field, and data management and processing.
- The University of Connecticut principal investigator for this application, Susan Reisine, Ph.D., is Associate Dean for Research in the School of Dental Medicine at the University of Connecticut Health Center. Her area of research focus has been on oral health disparities among low income and ethnic minorities in the USA.
- The ICR principal investigator for this project, Jean J. Schensul, Ph.D., is its founding director, dating back to 1987. Since 2005, she has been a Senior Scientist there; she has focused her research career on collaborative research to address health disparities in vulnerable populations. She has been a PI or Co-I for over 25 NIH-supported studies and supplements, and for a number of federal and private foundation studies.
- Dr. Reisine and Dr. Schensul have collaborated over the past five years to develop and implement two oral health projects funded by NIDCR, an R34 and an RC4.

Weaknesses

- Coordination between the University of Connecticut School of Dental Medicine and ICR will be required. Nevertheless, these entities and team have been working together for the past three years, with NIDCR funding, to conduct the R34 project and to build research infrastructure in housing for diverse and disabled older adults.

3. Innovation:

Strengths

- The application to promote oral health self management in older/disabled adults in urban, residential housing centers allows the project focus to be more population-based than individual-based.
- The inclusion of housing residents as partners in providing the pro-oral health campaigns is innovative and likely would have appeal to their counterparts.
- The research design bi-level, sequential approach (i.e., modified fractional factorial design) is an innovative alternative to traditional RCTs.

Weaknesses

- The ideas about promoting oral health self management are not in themselves new, although the combination of group- and individual-based interventions are interesting.
- There is little evidence for the validity and other psychometric properties of the new Dental Worries scale, other than a measure of internal consistency. The psychometric development of that scale is not described and may be based solely on the pilot project, without proper formal psychometric development.

4. Approach:

Strengths

- The intervention is theory based, allowing for testable hypotheses to be formulated.
- The programmatic nature of this research, following from a R34 project and a RC4 grant, is a strength of the proposed study.

- The proposed research design of this trial is strong, and is sensitive to environment (i.e., building-based stratification).
- Overall, the project has many strengths, and proposes to test the singular and joint effects of individually-based and group-based oral health promotion efforts.

Weaknesses

- There are no noted publications from the pilot work on which this U01 or the RC4 is based.
- Overall, the application is stronger and more heavily based on theory, while more practical, protocol-based information is needed in a sequential presentation with fewer acronyms for clarity.
- The application indicated that approximately 10% of residents cannot write at all. It is unclear whether residents will complete the surveys themselves on laptops, or if they will be interviewed. Regardless of which method is used, there are concerns about comprehension, and lack of testing for comprehensibility in the subjects.
- More details about the practice-to-mastery scoring are needed (e.g., anchors and examples that correspond with each of the points on the scale).
- Analyses should take into account the age of the resident, and the primary reason for their living in the building (i.e., older adult or handicapped status).
- The use of the “passport” at group events is unclear. Will the resident keep the passport and be expected to remember to bring it to each event? What happens if it is lost?
- The number of natural teeth required for inclusion appears to be one. Participants with one or very few teeth, however, would be difficult to assess with the primary and secondary endpoints, which may well be affected by number of extant teeth.
- It is unclear how residents might be “judged to be incompetent.” Use of some standard screening measure of cognitive functioning would be more ideal. Additionally, how will the investigators know which residents are “under conservatorship.” If based on the resident’s self-report, that would be highly suspect.

5. Environment:

Strengths

- The University of Connecticut School of Dental Medicine provides excellent administrative and scientific resources for the proposed study.

Weaknesses

- There may be an inherent weakness in the university-ICR arrangement if the investigators from the University of Connecticut are not involved in any first-hand way at the building sites. Being intimately involved in the data collection, and interacting with residents, undoubtedly would provide a rich source of data from which a true understanding of the impact and implications of the project would be possible, and from which future ideas could be generated.

Clinical Trial Documentation (Study protocol, Clinical Investigator's Brochure or equivalent, and MOP or equivalent):

- The study protocol provides the NIDCR template.
- The Manual of Procedures (MOP) provides highly detailed information but does not clearly explain the implementation of the interventions.
- A sequential approach in both documents would be helpful in understanding the procedures. A general overview of interventions, and who conducts them and how, could be followed by more detailed explanations.

Plans for Patient Recruitment/Retention:

- There are six urban buildings with apartments that will be the study sites; they will be paired by size and ethnic/linguistic breakdown

- There is a question about whether the buildings that were the sites of the pilot study will again be involved in the present study. There would be some overlap with prior subjects, who already were exposed to the proposed or similar interventions.
- Preliminary data successfully were collected in buildings that are similar to or part of the proposed performance site.
- There is a history of the University of Connecticut School of Dental Medicine and ICR cooperating in the R34 grant project, which involved the recruitment of residents such as those who would be involved in the proposed study.

Safety Monitoring:

- There is a Data and Safety Monitoring Plan that seems appropriate for the project.

Protections for Human Subjects: Acceptable Risks and/or Adequate Protections

- This project likely would not only be of benefit to society in terms of increased knowledge, but also has the potential to be of direct help to participants in terms of raising awareness about their personal oral health.

Data and Safety Monitoring Plan: Acceptable

  - A Data and Safety Monitoring Board (DSMB) is proposed for this study, with a detailed and well-articulated plan.

Inclusion of Women, Minorities and Children:
G1A - Both Genders, Acceptable
M1A - Minority and Non-minority, Acceptable
C1A - Children and Adults, Acceptable

Vertebrate Animals: Not Applicable (No Vertebrate Animals)

Biohazards: Not Applicable (No Biohazards)

Resource Sharing Plans: Unacceptable

Budget and Period of Support: Recommend as Requested

- No concerns.

Additional Comments to Applicant (Optional):

- In the application, a single general health status item is proposed. The investigators may wish to also consider including a single oral (dental) health status item that is seen elsewhere in the literature.
- The number of different acronyms in the application makes it difficult to follow.
- There are single item dental fear measures from established scales that could be usefully included without much additional burden.

CRITIQUE 4:

Significance: 2
Investigator(s): 1
Innovation: 2
Approach: 2
Environment: 1

Overall Impact: This clinical trial aims to study health behavior/health education methodology that has been previously employed in the medical field, in public health efforts and to a certain extent in dentistry. The use of a multilevel approach seems to be plausible and merits evaluation in an elderly population for enhancement of oral hygiene procedures.
Reviewers will consider each of the first five review criteria below in the determination of scientific and technical merit, and give a separate criterion score for each. Subsequent review criteria are factored into the Overall Impact score but do not receive an individual criterion score.

1. Significance:
Strengths
- Use of multilevel approach for motivation and support for the elderly segment of the population is significant as well as the analytical method for such approach (MFFD)

Weaknesses
- Some of the approaches proposed by this project have already been tested individually or with a multilevel approach, some successfully and others not.

2. Investigator(s):
Strengths
- Investigators are very well qualified and experienced as to attain the proposed aims

Weaknesses
- *[Not Addressed by Reviewer]*

3. Innovation:
Strengths
- A combination of methods (multilevel approach) employed for understanding and motivating oral health knowledge and changes is significant. Testing the sequence of approaches (individual x group campaigns and vice-versa) is worth noting.

Weaknesses
- Overall many of the health/behavior health/education methodologies have been around for quite a while and tested for that matter.

4. Approach:
Strengths
- Multilevel approach, testing for sequence of events and MFFD analysis

Weaknesses
- The primary outcomes are the Plaque and Gingival Indexes. The authors do not mention adjustments for the total number of teeth
- The Plaque Index reflects current brushing (or not) prior to examinations and that ia limitation of the index. As such participants will experience a learning curve that will likely influence the results of the study overtime. The Gingival Index would be a more realistic indication of study success although there are more sensitive methods such as the Papillary Bleeding Score.
- The level of gingival bleeding during the pilot study did not impress the reviewer. An average of 1 indicates minor gingival inflammation with no bleeding. The investigators should be aware of that while determining the impact of the trial
- In elderly populations medication intake can significantly affect oral health. No provisions were made to document medication intake
- Including individuals 40-62 years old with disabilities may skew some of the results and may not translate into recommendations for the elderly population in question

5. Environment:
Strengths

- Environment is appropriate

Weaknesses

- *[Not Addressed by Reviewer]*

Clinical Trial Documentation (Study protocol, Clinical Investigator's Brochure or equivalent, and MOP or equivalent):

- Appropriate

Plans for Patient Recruitment/Retention:

- Appropriate

Safety Monitoring:

- yes

Protections for Human Subjects: Acceptable Risks and/or Adequate Protections
Data and Safety Monitoring Plan: Acceptable

Inclusion of Women, Minorities and Children:
G1A - Both Genders, Acceptable
M1A - Minority and Non-minority, Acceptable
C1A - Children and Adults, Acceptable

Vertebrate Animals: Not Applicable (No Vertebrate Animals)

Biohazards: Not Applicable (No Biohazards)

Resource Sharing Plans: Acceptable

Budget and Period of Support: Recommend as Requested

CRITIQUE 5:

Significance:	3
Investigator(s):	1
Innovation:	2
Approach:	3
Environment:	1

Overall Impact: Overall this project addresses an important and timely issue about the management of dental disease prevention and control in the elderly and disabled patients who might not have adequate access to care. A clear and convincing argument is made for the importance of dental care in the overall health care of such patients, and the efficacy of specific intensive interventions in this process. This study is based on a successfully completed U01 pilot that demonstrates the potential for improvement in dental health with the implementation of the described interventions and the exploration of which interventions and in which order will best achieve the goals. The study is well designed with appropriate measures and only minor missing details. However, my enthusiasm is slightly reduced by the lack of any discussion about how such a program would be made affordable and available outside of a clinical study. The exact structure of the timing of all the training and data collection is not clearly diagramed or presented. The prevention of dropout discussion suggests an intensive involvement of staff over the entire course of the 14 months of data collection which does not mimic the more likely intermittent intervention that may be possible in a real clinical environment. There is no discussion about how the types of interventions planned in this trial would be possible in a real life setting. Without

this information it is difficult to know if the findings of this application will have any real effect on clinical care beyond intensive ongoing interventions.
Reviewers will consider each of the first five review criteria below in the determination of scientific and technical merit, and give a separate criterion score for each. Subsequent review criteria are factored into the Overall Impact score but do not receive an individual criterion score.

1. Significance:
Strengths
- Very strong demonstration of the need for improved dental health in the aging and disabled population.
- Clear description of the types of interventions that will be tested and rationale for them.

Weaknesses
- Lack of presentation of any studies of implementation of such interventions outside of clinical trials that would provide an understanding of how such interventions might be applicable to a “real world” environment

2. Investigator(s):
Strengths
- Very strong group of researchers with all the appropriate skills to carry out this trial

Weaknesses
- As discussed in the budget, it is unclear what the differences are in some of the roles. Drs. Douglas and Goldblatt appear to have very similar roles.

3. Innovation:
Strengths
1) This study is innovative in the broad extent of the interventions to be tested and the large number of measures and outcomes.

Weaknesses
1) While the extensive number of measures is a potential benefit, they also may be a burden to patients and will markedly increase the interactions between staff and patients that may produce improved outcomes based on the extent of personal interaction beyond the planned interventions.

4. Approach:
Strengths
- The procedures and measures are carefully explained. Although a confusing mix of multiple approaches, the underlying principles of the primary study are understandable.

Weaknesses
- The approach has a potential significant weakness. The investigators do not provide (or at least this reviewer could not find) a simple and understandable table or description of the amount of interaction between interventionalist and patients. It is not clear how long each intervention is planned (how many days will a patient be involved in interaction with the interventionalist and over what period) followed by what period of observation, and with what frequency. Given the 1.5 FTE of time for the interventionalist, with 150 patients in each 14 month sequence, this is approximately 30 minutes a week per patient. Clarity on this issue is important to adequately understand the intervention and the potential for implementation in a broader population.

5. Environment:
Strengths
- The two institutions involved in this application have extensive experience with this type of research and have adequate access to all necessary resources

Weaknesses

- None to speak of

Clinical Trial Documentation (Study protocol, Clinical Investigator's Brochure or equivalent, and MOP or equivalent):

- Provided but is again missing a simple description or diagram of the activities of the proposed project.

Plans for Patient Recruitment/Retention:

- Appropriate with issues as discussed above

Safety Monitoring:

- Appropriate

Protections for Human Subjects: Acceptable Risks and/or Adequate Protections
Data and Safety Monitoring Plan: Acceptable

Inclusion of Women, Minorities and Children:
G1A - Both Genders, Acceptable
M1A - Minority and Non-minority, Acceptable
C1A - Children and Adults, Acceptable

- Appropriate for the issue being studied, namely elderly and disabled adults. Children pose a separate problem that is likely to be very different.

Vertebrate Animals: Not Applicable (No Vertebrate Animals)

Biohazards: Not Applicable (No Biohazards)

Resource Sharing Plans: Acceptable

Budget and Period of Support:
Recommended budget modifications or possible overlap identified:

- Unclear about the distinction in roles of some investigators included and the subaward budget needs a justification for the amount of time requested for all staff

Additional Comments to Applicant (Optional):

- In the discussion of this application it seems that an underlying concept for this study is that the community intervention will have a better long term effect on oral health than the individual counseling. What is not clearly stated is how long the intervention will take place and how long after the intervention the measures will be in place to assess ongoing effect. Also, it is unclear how often measurements of the outcome will be undertaken. If the intervention is ongoing, then there is a serious concern about the implementation of interventions in any real world setting. This must be clarified.

CRITIQUE 6:

Significance:	2
Investigator(s):	2
Innovation:	2
Approach:	4
Environment:	2

Overall Impact: This study employs a modified fractional factorial design (MFFD) as an alternative to an RCT. It has the important aim of identifying means of improving oral health in older adults. While the MFFD is innovative, it has the disadvantage of lacking a control group with no intervention. However, each subject's baseline status serves as his/her own control and one might suppose that the absence of intervention could hardly be expected to improve oral health. In this design, the randomization is done at the level of the building of residence, and the two interventions are randomly given in one order or the other. The ability to compare the two interventions (individual-level AMI-PM and building-level CA-PM) after the first period, and to later see how they work in combination is appealing. The outcomes are well-defined and a detailed analytic plan is presented. In power and sample size considerations, the authors take a conservative approach (e.g., using a smaller effect size than observed in pilot data and the upper CL of the variance estimate) to ensure adequate power for important effects, which is a strength.

Protections for Human Subjects: Acceptable Risks and/or Adequate Protections
Data and Safety Monitoring Plan: Acceptable

Inclusion of Women, Minorities and Children:
G1A - Both Genders, Acceptable
M1A - Minority and Non-minority, Acceptable
C1A - Children and Adults, Acceptable

Vertebrate Animals: Not Applicable (No Vertebrate Animals)

Biohazards: Not Applicable (No Biohazards)

Budget and Period of Support:
- *[Not Addressed by Reviewer]*

CRITIQUE 7:

Significance:	2
Investigator(s):	2
Innovation:	3
Approach:	3
Environment:	3

Overall Impact: This application has the potential to make a great impact on the field of oral health prevention. It proposes a unique intervention project that integrates peer education, activities, plus individual preventive behavior to improve oral health, in a community with accumulated oral disease. The study involves two vulnerable populations: a minority older adult population and adults with disabilities; it represents a very strong, well-designed study, with a capable multidisciplinary team, that can inform the research community on a number of levels.

1. Significance:
Strengths
- The proposed study's significance lies in the potential to study a complex intervention that integrates two levels (the individual and the group) within a much-neglected community-dwelling population (older, minority, low income adults and adults with disabilities).
- The study has the potential to improve oral health norms among this population by developing a methodology for preventive interventions in elders' residence offered in conjunction with peers,

but formed by oral health professionals who translate research findings and provide the input through lay language FAQs and educational games and events.
- The study offers the ability to evaluate the intervention in such as way as to tease out the nuances of the intervention's success in using Theory of Reasoned Action, Theory of Planned Behavior and the Integrative Model of Behavioral Prediction.
- A critical barrier has been the paucity of multi-site studies on oral health behavior and the use of single populations. This study brings together several years of careful positioning that created infrastructure for studying individual oral health behavior, how to provide information to older adults and younger adults with disabilities both of which reside in low income housing, and of which some will be selected as 'peer leaders' to participate in the building-level campaign. These components are significant because it has a great potential for understanding how an individual changes behavior and how medical directors could use peer leaders to effect behavior change within group residences.

Weaknesses
- This is a very strong application and the Weaknesses are few. One may be that, while the investigators have a very strong application to assess all of these components described above, this is a very complex study and it will be challenging to keep all of the balls in the air and not allow once section to fail. A stronger section describing how they will manage the integration of the components might have been useful.

2. Investigator(s):
Strengths
- The investigators are very well suited to the project and bring many years of successful behavioral and community-based research.
- Dr. Susan Reisine is Professor and Associate Dean of the U Conn School of Dental Medicine, is an internationally known sociologist who is a leader in behavioral research in oral health.
- Dr. Jean Schensul, founding director of ICR, is a well-recognized methodologist with many years of experience in community-based epidemiologic, qualitative and intervention research. They successfully collaborated on the infrastructure and intervention pilots.
- The surrounding team includes: Dr. Joanna Douglass, a dentist with experience in community-based intervention research and who will provide oral health expertise, as well as input on design and oversight of the clinical assessments and interpretation. Dr. Ruth Goldblatt is a geriatric dentist and recognized oral health educator and Associate Clinical Professor and Director of Dentistry at Hebrew Home and Hospital. She has guided dental examiners in the clinical assessments on the pilot study and will continue to do so. Kim Radda, RN and MA is a nurse anthropologist with many years of experience in research with older adults. She guides the field operations and data interpretation.

Weaknesses
- A possible weakness is that none of the Key Personnel were identified as experts in AMI or developing oral health education public campaigns such as these. The addition of a adult education specialist could be valuable. In fact, they had such expertise on the pilot(highly experienced bilingual senior research educators), but seem to have not included them in the large scale investigation. This is an easily fixed, nonfatal flaw.

3. Innovation:
Strengths
- The use of the Modified Fractional Factorial Design to evaluate components of the intervention; use of baseline data to guide tailoring of AMI-PM and sample size calculation;
- The enhancement of a community-based oral health intervention with a peer building-level education campaign with events to deliver targeted education;

- developing a means of tailoring the adapted motivational interviewing intervention in different environmental contexts;
- The use of clinical measures in a community setting for diagnostic, educational, motivational and evaluation purposes;

Weaknesses

The investigators have incorporated the development and use of a new scale to assess oral health fears in the target population, but existing scales already exist and not clear why they chose to do so.

4. Approach:
Strengths

- Clinical research study is carefully guided by Fishbein's noted theory for reasoned action & operationalized by Adapted Motivational Interviewing.
- A comprehensive model that is shaped by the literature.
- Bilevel approach with both individual and group **interventions**;
- Offers dual **outcomes** – oral hygiene behaviors (practice) and oral health clinical (GI, PI) and psychosocial/QOL GOHAI.
- A complex analysis plan that evaluates and will inform the investigators on what portion of intervention worked, and which order of intervention worked best.

Weaknesses

- IN the pilot data, they reported that brushing scores increased from 1.9 (fair/poor) to 2.9(good/excellent) after the AMI-PM session and flossing increases were similar. In the study methods there in no masking of the investigators conducting the clinical evaluations (They know which time period and thus are not blinded.) It is possible to imagine that the investigators might be biased knowing the adults had training.

5. Environment:
Strengths

- The environment contributes greatly to the potential for success. The application has been submitted by two institutions who have worked together for five years, Univ. Conneticut SOD with over $10.5 million in extramural funding and the Institute for Community Resaerch, a 25 year old non-profit research institute that has worked with on community-based projects in a number of areas, including health and mental health research with low income adults, and drug abuse, and AIDS studies.
- The application uses the Multiple PI (Susan Resine and Jean Schensul) and both investigators are highly experienced in oral health research and have collaborated on two successful NIDCR funded grants.
- The investigators have successfully completed a pilot study and incorporated lessons learned on how to establish entry into low income senior homes, getting to know the building management, conducting presentations in English and Spanish, etc. into this application.

Weaknesses

- The Connecticut Task Force on Oral Health for Older Adults, which includes the Oral Health Research Strategic Alliance and the North Central Area Agency on Aging were also mentioned as taking part, but I could not find information describing their role in the methods.

Protections for Human Subjects: Acceptable Risks and/or Adequate Protections

- The risks for this study are minimal.
- The investigators have excluded people who might have a biological risk to subacute bacterial endocarditis;
- The intervention is educational, and psychological.

Data and Safety Monitoring Plan (Applicable for Clinical Trials Only):

Inclusion of Women, Minorities and Children:
G1A - Both Genders, Acceptable
M1A - Minority and Non-minority, Acceptable
C1A - Children and Adults, Acceptable

- The intervention takes place in senior housing, which also accepts disabled adults. Thus, children 18-20 may be found in the included target area.

Vertebrate Animals: Not Applicable (No Vertebrate Animals)

Biohazards: Not Applicable (No Biohazards)

Budget and Period of Support:

- *[Not Addressed by Reviewer]*

THE FOLLOWING RESUME SECTIONS WERE PREPARED BY THE SCIENTIFIC REVIEW OFFICER TO SUMMARIZE THE OUTCOME OF DISCUSSIONS OF THE REVIEW COMMITTEE ON THE FOLLOWING ISSUES:

PROTECTIONS FOR HUMAN SUBJECTS (Resume): ACCEPTABLE

INCLUSION OF WOMEN PLAN (Resume): ACCEPTABLE

INCLUSION OF MINORITIES PLAN (Resume): ACCEPTABLE

INCLUSION OF CHILDREN PLAN (Resume): ACCEPTABLE

SCIENTIFIC REVIEW OFFICER'S NOTES: Although the protections of human subjects plan is adequate, given the literacy levels of the subjects and potential competency issues for residents under receivership, additional protections may be necessary.

COMMITTEE BUDGET RECOMMENDATIONS: The budget was recommended as requested.

NIH has modified its policy regarding the receipt of resubmissions (amended applications). See Guide Notice NOT-OD-10-080 at http://grants.nih.gov/grants/guide/notice-files/NOT-OD-10-080.html.
The impact/priority score is calculated after discussion of an application by averaging the overall scores (1-9) given by all voting reviewers on the committee and multiplying by 10. The criterion scores are submitted prior to the meeting by the individual reviewers assigned to an application, and are not discussed specifically at the review meeting or calculated into the overall impact score. Some applications also receive a percentile ranking. For details on the review process, see http://grants.nih.gov/grants/peer_review_process.htm#scoring.

MEETING ROSTER

National Institute of Dental and Craniofacial Research Special Emphasis Panel
NATIONAL INSTITUTE OF DENTAL & CRANIOFACIAL RESEARCH
Clinical Trial or Biomarker Clinical Evaluation Study Planning Grant and Clinical Trial Implementation or Biomarker Clinical Evaluation Study Cooperative Agreement Applications Review Panel
ZDE1 VH (22) 1
November 06, 2013

CHAIRPERSON

MCNEIL, DANIEL W., PHD
PROFESSOR
DEPARTMENT OF PSYCHOLOGY
CLINICAL PROFESSOR, DEPT OF DENTAL PRACTICE
AND RURAL HEALTH
WEST VIRGINIA UNIVERSITY
MORGANTOWN, WV 26506

MEMBERS

ATCHISON, KATHRYN ANN, DDS
PROFESSOR & ASSOCIATE DEAN FOR RESEARCH &
KNOWLEDGE MANAGEMENT
OFFICE OF INTELLECTUAL PROPERTY AND INDUSTRY
SPONSORED RESEARCH
UNIVERSITY OF CALIFORNIA, LOS ANGELES
LOS ANGELES, CA 90095

BRETZ, WALTER A. BRETZ, DDS, DPH
ASSOCIATE PROFESSOR
DIVISION OF PEDIATRIC AND DEVELOPMENTAL
SCIENCES
SCHOOL OF DENTAL MEDICINE
UNIVERSITY OF PITTSBURGH
PITTSBURGH, PA 15261

CARLSON, CHARLES RUSSELL, PHD
PROFESSOR & CHAIR
DEPTARTMENT OF PSYCHOLOGY
UNIVERSITY OF KENTUCKY
LEXINGTON, KY 40506

DOUCETTE, JOHN THOMAS, PHD
ASSISTANT PROFESSOR
DEPARTMENT OF PREVENTIVE MEDICINE
SCHOOL OF MEDICINE
MOUNT SINAI
NEW YORK, NY 10029

FARRAR, JOHN T, MD, PHD
SENIOR SCHOLAR
DEPARTMENT OF BIOSTATISTICS AND EPIDEMIOLOGY
CENTER FOR CLINICAL EPIDEMIOLOGY
AND BIOSTATISTICS
UNIVERSITY OF PENNSYLVANIA SCHOOL OF MEDICINE
PHILADELPHIA, PA 19104

GLAROS, ALAN G., PHD
ASSOCIATE DEAN & PROFESSOR
DIVISION OF BASIC BIOMEDICAL SCIENCES
COLLEGE OF OSTEOPATHIC MEDICINE
KANSAS CITY UNIVERSITY OF
MEDICINE AND BIOSCIENCES
KANSAS CITY, MO 64106

HARVEY-BERINO, JEAN R, PHD
PROFESSOR AND CHAIR
DEPARTMENT OF NUTRITION
AND FOOD SCIENCES
UNIVERSITY OF VERMONT
BURLINGTON, VT 05405

MULLER, KEITH E, PHD
PROFESSOR
STATISTICS AND DATA CORE
DEPARTMENT OF HEALTH OUTCOMES AND POLICY
COLLEGE OF MEDICINE
UNIVERSITY OF FLORIDA
GAINESVILLE, FL 32608

RUSH, WILLIAM ADAMS PHD, PHD
SENIOR PROGRAMMER AND ANALYST
HEALTH PARTNERS RESEARCH FOUNDATION
MINNEAPOLIS, MN 55440

SHIELDS, CLEVELAND GEORGE, PHD
DEPARTMENT OF CHILD DEVELOPMENT AND
FAMILY STUDIES
PURDUE UNIVERSITY
WEST LAFAYETTE, IN 47907

SOHN, WOOSUNG , DDS, PHD, DRPH
ASSISTANT PROFESSOR
DEPT OF CARIOLOGY/RESTORATIVE SCIENCES;
ENDODONTIC
SCHOOL OF DENTISTRY
UNIVERSITY OF MICHIGAN
ANN ARBOR, MI 481091078

WALJI, MUHAMMAD , PHD
DEPARTMENT OF DIAGNOSTIC SCIENCES
UNIVERSITY OF TEXAS DENTAL BRANCH AT HOUSTON
ADJUNCT ASSISTANT PROFESSOR
UNIVERSITY OF TEXAS SCHOOL OF HEALTH
INFORMATION
HOUSTON, TX 77030

WEWERS, MARY ELLEN, RN, PHD
PROFESSOR
DIVISION OF EPIDEMIOLOGY
COLLEGE OF PUBLIC HEALTH
THE OHIO STATE UNIVERSITY
COLUMBUS, OH 432101289

SCIENTIFIC REVIEW ADMINISTRATOR

HENRIQUEZ, VICTOR , PHD
SCIENTIFIC REVIEW OFFICER
SCIENTIFIC REVIEW BRANCH
DIVISION OF EXTRAMURAL ACTIVITIES
NATL INSTITUTE OF DENTAL AND CRANIOFACIAL
RESEARCH
NATIONAL INSTITUTES OF HEALTH
BETHESDA, MD 20892

GRANTS TECHNICAL ASSISTANT

FAKINLEDE, STACEY , BS
PROGRAM SPECIALIST
SCIENTIFIC REVIEW BRANCH
DIVISION OF EXTRAMURAL ACTIVITIES
NATL INSTITUTE OF DENTAL AND CRANIOFACIAL RESEARCH
NATIONAL INSTITUTES OF HEALTH
BETHESDA, MD 20892

Consultants are required to absent themselves from the room during the review of any application if their presence would constitute or appear to constitute a conflict of interest.
